# Lichen biomonitoring to assess spatial variability, potential sources and human health risks of polycyclic aromatic hydrocarbons (PAHs) and airborne metal concentrations in Manchester (UK)

**DOI:** 10.1007/s10661-024-12522-4

**Published:** 2024-03-18

**Authors:** Daniel Niepsch, Leon J. Clarke, Rhys G. Jones, Konstantinos Tzoulas, Gina Cavan

**Affiliations:** 1 Department of Natural Sciences, Faculty of Science and Engineering, Manchester Metropolitan University, Manchester, M1 5GD UK; 2 Waters Corporation, Wilmslow, SK9 4AX UK

**Keywords:** Persistent organic pollutants, Air quality assessment, GC-APCI-MS/MS, Human health, Biomonitor

## Abstract

Airborne metals and organic pollutants are linked to severe human health impacts, i.e. affecting the nervous system and being associated with cancer. Airborne metals and polycyclic aromatic hydrocarbons (PAHs) in urban environments are derived from diverse sources, including combustion and industrial and vehicular emissions, posing a threat to air quality and subsequently human health. A lichen biomonitoring approach was used to assess spatial variability of airborne metals and PAHs, identify potential pollution sources and assess human health risks across the City of Manchester (UK). Metal concentrations recorded in lichen samples were highest within the city centre area and along the major road network, and lichen PAH profiles were dominated by 4-ring PAHs (189.82 ng g^−1^ in *Xanthoria parietina*), with 5- and 6-ring PAHs also contributing to the overall PAH profile. Cluster analysis and pollution index factor (PIF) calculations for lichen-derived metal concentrations suggested deteriorated air quality being primarily linked to vehicular emissions. Comparably, PAH diagnostic ratios identified vehicular sources as a primary cause of PAH pollution across Manchester. However, local more complex sources (e.g. industrial emissions) were further identified. Human health risk assessment found a “moderate” risk for adults and children by airborne potential harmful element (PHEs) concentrations, whereas PAH exposure in Manchester is potentially linked to 1455 (ILCR = 1.45 × 10^−3^) cancer cases (in 1,000,000). Findings of this study indicate that an easy-to-use lichen biomonitoring approach can aid to identify hotspots of impaired air quality and potential human health impacts by airborne metals and PAHs across an urban environment, particularly at locations that are not continuously covered by (non-)automated air quality measurement programmes.

**Graphical Abstract:**

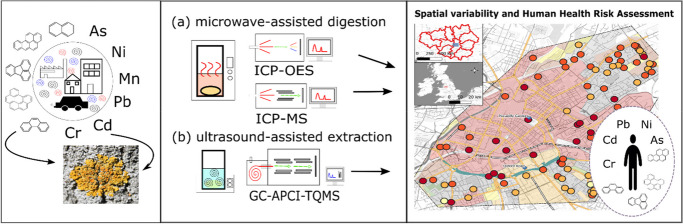

**Supplementary Information:**

The online version contains supplementary material available at 10.1007/s10661-024-12522-4.

## Introduction

Atmospheric pollution in urban areas has major impacts on human health (Gulia et al., 2015). For instance, airborne metals (e.g. arsenic [As], chromium [Cr], cadmium [Cd], iron [Fe], lead [Pb] and zinc [Zn]) can have toxic impacts on human health, particularly affecting the urinary and nervous system and are potentially linked to Alzheimer’s disease (Jaishankar et al., 2014; Kampa & Castanas, 2008; Maher et al., 2016; Morais et al., 2012). Organic contaminants such as polycyclic aromatic hydrocarbons (PAHs) are shown to be carcinogenic, especially lung, bladder and liver cancers and are considered ubiquitous, persistent and highly lipo-soluble (i.e. able to accumulate) in the environment (Augusto et al., 2016; International Agency for Research on Cancer (IARC), 2014).

Airborne metals in urban environments are derived from natural sources (e.g. geochemical sources) and anthropogenic sources, e.g. combustion, industrial and manufacturing, as well as vehicle exhaust emissions, tyre and body wear and break lining material (Kampa & Castanas, 2008; Taylor, 2006; Taylor & Robertson, 2009). Comparably, PAHs in urban environments are connected to anthropogenic sources, e.g. heating, industrial processes (i.e. chemical manufacturing) and transportation/road traffic (Augusto et al., 2015; UBA, 2016).

Potentially harmful elements (PHEs) include metals and metalloids (e.g. Ag, As, Be, Cd, Cr, Cu, Hg, Ni, Pb, Sb, Se, Th and Zn) and 16 PAHs of primary interest that pose adverse human health impacts, which have been identified by the U.S. Environmental Protection Agency (EPA, 1999, 2014; Lerda, 2011). The latter include naphthalene, acenaphthylene, acenaphthene, fluorene, phenanthrene, anthracene, fluoranthene, pyrene, benz[a]anthracene, chrysene, benzo[b]fluoranthene, benzo[k]fluoranthene, benzo[a]pyrene, indeno[1,2,3-cd]pyrene, dibenz[a,h]anthracene and benzo[ghi]perylene (EPA, 1999; Lerda, 2011), and for seven of those priority PAHs, their toxicity has been recognised, e.g. benzo[a]pyrene being one of the most toxic PAHs (Augusto et al., 2015; Domínguez-Morueco et al., 2015). The UKs ‘Polycyclic Aromatic Hydrocarbon Network’ currently monitors ambient PAH concentrations at 31 sites across the country (e.g. Edinburgh, Liverpool, London and Salford/Eccles; (DEFRA, 2014a), including the 16 EPA priority PAHs. However, PAH measurements in urban areas are only undertaken by a locally limited number of automated and non-automated monitoring stations, not providing spatial variability of concentrations across urban environments. Therefore, it would be advantageous to apply additional monitoring methods to assess variability of airborne metal and PAH concentrations that could indicate poor air quality and subsequent human health impacts across urban environments.

Lichens have been extensively used for biomonitoring studies and as ecological indicators for air pollution, in particular, where costly technical equipment cannot be afforded or is not viable (Forbes, 2015; Van der Wat & Forbes, 2015). Because lichens readily accumulate pollutants within their thallus, even when present at low concentrations, they have been widely considered as reliable biomonitors for atmospheric (metal) pollution around the world (e.g. Italy, Spain, Turkey, Bosnia and Herzegovina, Malaysia and Thailand; Abas et al., 2019, Abas et al., 2020; Abas, 2021; Boonpeng et al., 2023; Bozkurt, 2017; Giordani et al., 2012; Kularatne & De Freitas, 2013; L. Paoli et al., 2012; Parviainen et al., 2019; Ramić et al., 2019). Other biomonitors, e.g. tree compartments (e.g. bark, leaves and branches) have been widely used to monitor periods and sources of metal pollution (Ateya et al., 2023; Cobanoglu et al., 2023; Forbes et al., 2015; Isinkaralar et al., 2022; Key et al., 2022, 2023; Koç, 2021) that may support a lichen biomonitoring approach to further identify intervals of (elevated) pollution and recognise pollution sources. However, lichen tend to equilibrate with their surrounding environment and reply fast under deteriorating conditions (e.g. metal concentrations; Paoli et al., 2018a, 2018b), and young lichen specimen was targeted in this study to assess recent spatio-temporal variability of air quality.

Due to recent interest in persistent organic pollutants in the environment, numerous PAH-related lichen studies have been undertaken in urban areas and to monitor traffic pollution around the world (e.g. India, Italy, Spain, Portugal and France) either using native species or transplanted lichens (Augusto et al., 2010; Blasco et al., 2006; Domeño et al., 2006; Guidotti et al., 2009; Kodnik et al., 2015; Nascimbene et al., 2014; Owczarek et al., 2001; Shukla & Upreti, 2009; Shukla et al., 2012). Whilst a lot of PAH-related biomonitoring studies were undertaken around the world, only one study focused on an urban area (London) in the UK, using transplants of the lichen *Pseudevernia furfuracea* (Vingiani et al., 2015). This work will illustrate the potential to apply such an easy-to-use and cost-effective approach to identify areas of poor air quality and human health concern, inform about the necessity of additional urban air quality monitoring programmes and to assess and compare airborne metal and PAH levels across urban environments in the UK (and other countries).

This is the first study using a lichen biomonitoring approach to evaluate spatial variability of airborne metal and PAH pollution in the City of Manchester (UK). Metal concentrations (i.e. PHEs such as As, Cd, Cr Ni, Mn and Pb) and the 16 EPA priority PAH concentrations were further used to identify potential pollution sources by applying ‘pollution index factors’ (PIF; Boamponsem et al., 2010; Bozkurt, 2017) and PAH diagnostic ratios. Moreover, human health risks were assessed using ‘average daily dose (ADD)’ and the ‘hazard index (HI)’ for PHEs ((EPA, 1989, 1996; Khodadadi et al., 2023; van den Berg, 1994), and ‘toxic equivalence factors’ (TEFs) and ‘incremental lifetime cancer risk’ (ILCR) using lichen-derived PAH concentrations (Augusto et al., 2013; EPA, 1993, 2005; Nisbet & LaGoy, 1992).

## Materials and methods

### Study area—the City of Manchester (UK)

**Fig. 1:**
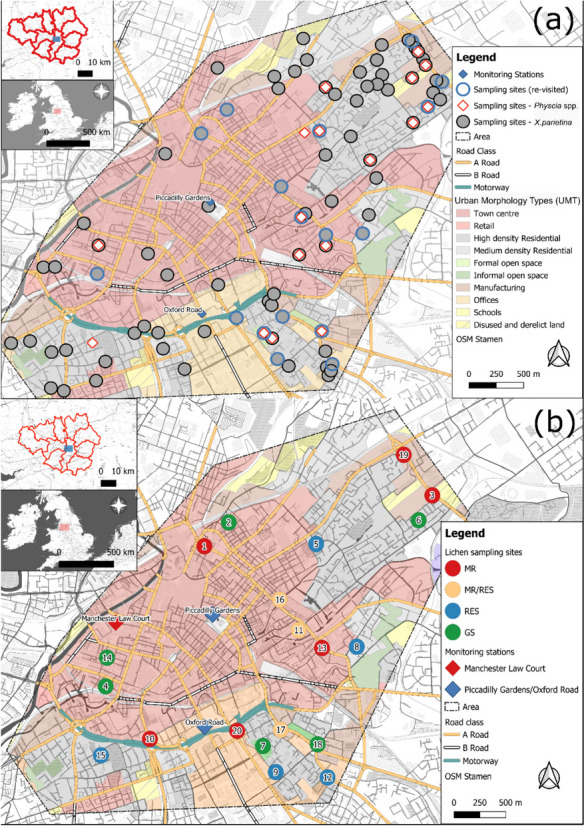
Lichen sampling sites for analysis of (**a**) airborne metal concentrations in *X. parietina* (*N* = 94) and *Physcia* spp. (*N* = 19) and (**b**) sampling locations (*X. parietina*: *N* = 20 and *Physcia* spp.: *N* = 3) within the city centre of Manchester (displayed with site-ID), automated monitoring stations (including automated PAH Andersen sampler) are also shown. For (**b**) lichen sampling sites were classified with regard to their locations surrounding and potential higher and lower PAH concentrations. MR, major road (includes A-, B-roads and motorways); MR/RES, major road and residential; RES, residential; GS, greenspace; displayed with urban morphology types (UMTs)

The City of Manchester, as the centre of the Greater Manchester conurbation is the second largest built-up area located in the Northwest of England with 567,000 inhabitants (Greater Manchester: 2.7 million; Manchester City Council, 2019). Within its centre, two automated air quality monitoring stations (at Manchester Piccadilly Gardens and at Oxford Road; Fig. 1) record ambient concentrations of gaseous (e.g. NO_x_) and particulate (e.g. PM_2.5_/_10_) atmospheric pollutants, revealing problematic air quality and associated human health problems (Regan, 2018). For instance, Manchester is ranked highest in premature deaths regarding cancer in England (1.6 times higher compared to national average; Manchester City Council, 2017), suggesting potential health impacts linked to poor air quality, i.e. from airborne metals and PAHs.

No data for airborne metal concentrations is recorded at either automated air quality monitoring station, but elevated metal concentrations (e.g. lead and iron) have been reported in urban road dust/sediment samples within Manchester city centre (Robertson et al., 2003). Until 2014, PAHs were recorded at ‘Manchester Law Courts’ (Easting/Northing: 383,375, 398,260; UK-AIR ID: UKA00185) by automated high-volume Andersen (GPS-1) (DEFRA, 2014b). Since 2007, 27 PAHs in Greater Manchester are monitored at an urban background site ‘Salford Eccles’ (Easting/Northing: 377,925, 398,729; UK-Air ID: UKA00339) using a solid phase PAH Digitel DHA-80 particulate sampler, reporting a general trend (i.e. seasonal variability) of PAHs at this site (Conolly & Carpenter, 2021). Overall, the UKs primary sources of PAHs changed since 1991, i.e. a transition from industrial processes to emissions (65% of PAH emissions) dominated from transportation/road traffic (in 2005; Meijer et al., 2008). In contrast, due to the increase in the use of wood as domestic fuel, benzo[a]pyrene, a known carcinogen, is linked to residential and commercial combustion sources (Conolly & Carpenter, 2021; DEFRA, 2019). However, since discontinuation of Manchester’s city centre-based PAH monitoring programme, no data is available, demonstrating the necessity for contemporary monitoring and assessment of PAHs and impacts on urban air quality.

### Lichen sampling and processing

The research area focussed on the city centre area on a SW – NE transect across the city centre, to include the areas regularly monitored (by automated stations) for specific pollutants (e.g. NO_x_) and because potentially high levels of airborne metals and PAHs were expected.

Lichen samples were obtained from twigs and small branches of street trees across Manchester city centre (Fig. 1). Sampling locations were informed based on tree species, tree abundance, site accessibility and visible lichen growth on twigs and branches. Twigs and smaller branches were sampled between 2- and 4-m height (using a tree pruner) to obtain younger lichen specimen and to assess recent atmospheric pollution. Depending on lichen coverage (and accessibility) on individual trees, one or more cardinal directions (clockwise rotation, from facing the major road) were sampled, and lichen samples were combined into a single sample. Potential lichen sampling locations within the city centre area (Fig. 1) were limited, due to low tree density, and it was not possible to sample lichens from one tree species only, because of diverse ornamental and planted trees within the city centre; however, tree species (e.g. *Acer* sp., *Fraxinus* sp. and *Tilia* sp.) with similar bark acidity (Kirschbaum & Wirth, 2010) were sampled for lichens. Lichen species *X. parietina* and *Physcia* spp. (here combined as *Ph. adscendens* and *Ph. tenella*) are widely distributed, thrive in nitrogen-rich environments (i.e. nitrophytic) and are ubiquitously found in urban environments (Dobson, 2011; Kirschbaum & Wirth, 2010). Hence, providing ideal specimens for a biomonitoring approach, different lichen species were chosen to investigate species-specific differences in pollutant uptake and to investigate their accumulation potential and impacts by pollutants on their vitality (Augusto et al., 2015; Garty, 2001).

For elemental analysis, *X. parietina* (*N* = 94) and *Physcia* spp. (*N* = 17) were sampled from street trees across Manchester during dry days between June 2016 and October 2017 (Fig. 1a). Samples were placed in paper bags, transported to the lab and carefully scraped off the tree material under an illuminated magnifying glass, avoiding bark and other detritus, using a stainless-steel scalpel. Lichen material was ground using an agate pestle and mortar, and homogenized lichen powder was stored in glass vials at room temperature (20 °C) in the dark, away from chemicals.

For PAH analysis, lichen sampling sites (Fig. 1b) were informed based on lichen chemical data, i.e. nitrogen contents (wt%; Niepsch et al., 2023) and metal concentrations (µg g^−1^), in particular potentially harmful elements (PHEs, e.g. Cd, Cr, Ni, Mn, Pb and Zn; Table S1). *X. parietina* (*N* = 20) and *Physcia* spp. (*N* = 3) were sampled from street trees from different land-use patterns, e.g. residential, green spaces and major roads (Fig. 1b) between May 2018 and September 2018. *X. parietina* and *Physcia adscendens* have been used in environmental PAH pollution studies in Spain, Portugal and Poland (Augusto et al., 2016, 2015; Blasco et al., 2006; Owczarek et al., 2001), making these lichen species suitable for analysis of spatial variability of PAH concentrations. To minimise the potential loss of (volatile) organic pollutants, lichen samples were placed in paper bags, rapidly transported to the laboratory and processed on the same day (i.e. scraping off bark material using a stainless-steel scalpel) freeze dried on a ‘Büchi L-200’ overnight (for 12 h at − 55 °C; 0.03 mbar), subsequently homogenised using an agate pestle and mortar and stored in pre-fired (at 400 °C for 3 h) glass vials and freezer stored (at − 18 °C) until extraction and analysis (Blasco et al., 2006; Domeño et al., 2006; Forbes et al., 2015; Guidotti et al., 2009).

### Chemical extraction and instrumental analysis for metal concentrations

Microwave-assisted nitric acid (HNO_3_) digestion and analysis by ICP-OES and ICP-MS were used to determine lichen metal contents (based on Doǧrul Demiray et al. (2012) and amended to in-house capabilities). Microwave digestion vessels (PTFE) were pre-cleaned (with 7 mL of HNO_3_; 69% VWR Aristar® grade and 7 mL of ultrapure water; 18.2 MΩ), microwave processed (CEM Mars Xpress5), thoroughly rinsed with ultrapure water and oven dried at 80 °C prior to lichen material digestion.

About 0.25 g of ground and homogenised lichen sample powder (*X. parietina* and *Physcia* spp.) were weighed into pre-cleaned digestion vessels, followed by the addition of 2 mL ultrapure water and 8 mL of HNO_3_ (VWR Aristar grade®) and subsequent microwave digestion (digestion programme detailed in Table S2). Acid digestion solutions were gravity filtered into 50-mL volumetric flasks using Whatman® (grad 540, hardened ashless, 110 mm; Sigma-Aldrich) filter papers and made up to volume with ultrapure water. Solutions were decanted into 50-mL metal-free centrifuge tubes (VWR) and stored in the dark until determination of 12 elements by ICP-OES (Thermo Scientific iCap 6000 series) and 18 elements by ICP-MS (Agilent 7900). Both analytical techniques were used, due to advantages for each instrument, i.e. higher sensitivity and lower background signals (i.e. able to achieve parts-per-trillion detection limits) for ICP-MS, but higher sample throughput for ICP-OES (Agilent Technologies, 2006).

Selection of elements to analyse was based on the U.S. EPA list of priority substances and metals recorded in soils of the European Union (EPA, 2014; Tóth et al., 2016), as well as potential human health implications (e.g. Pb and Cd) and for source identification, e.g. vehicular emissions (i.e. Cr, Mn, Ni, Pd, Pt and Zn) and geological sources (e.g. Al, As and Fe). Based on polyatomic interferences of elements (Esslab, 2017; May et al., 1998) and instrument sensitivity, a decision matrix (Table S3) was used which metal to report for analytical instruments.

### Chemical extraction and instrumental analysis for PAHs

About 0.2 g of ground lichen material were weighed into fired (at 400 °C for 3 h) and solvent (Dichloromethane – DCM and Hexane – HEX) cleaned glass vials. All solvents were of HPLC grade (dichloromethane – DCM, ≥ 99.8% HiPerSolv Chromanorm® for HPCL by VWR; n-hexane—HEX ≈95% for HPLC by Fisher Scientific). Lichen material was spiked with 10 µL of 0.5 ng µL^−1^ deuterated PAH standard each prior to extraction, i.e. *phenanthrene d-10*, *chrysene-d12* and *dibenzo[a,h]anthracene-d14* (Cambridge Isotope Laboratories, Inc.; LGC standards, UK). Deuterated standards were used as relative internal standard for calculation of 16 priority PAHs (“Quality assurance and quality control (QA/QC) – GC–MS” section).

**Fig. 2:**
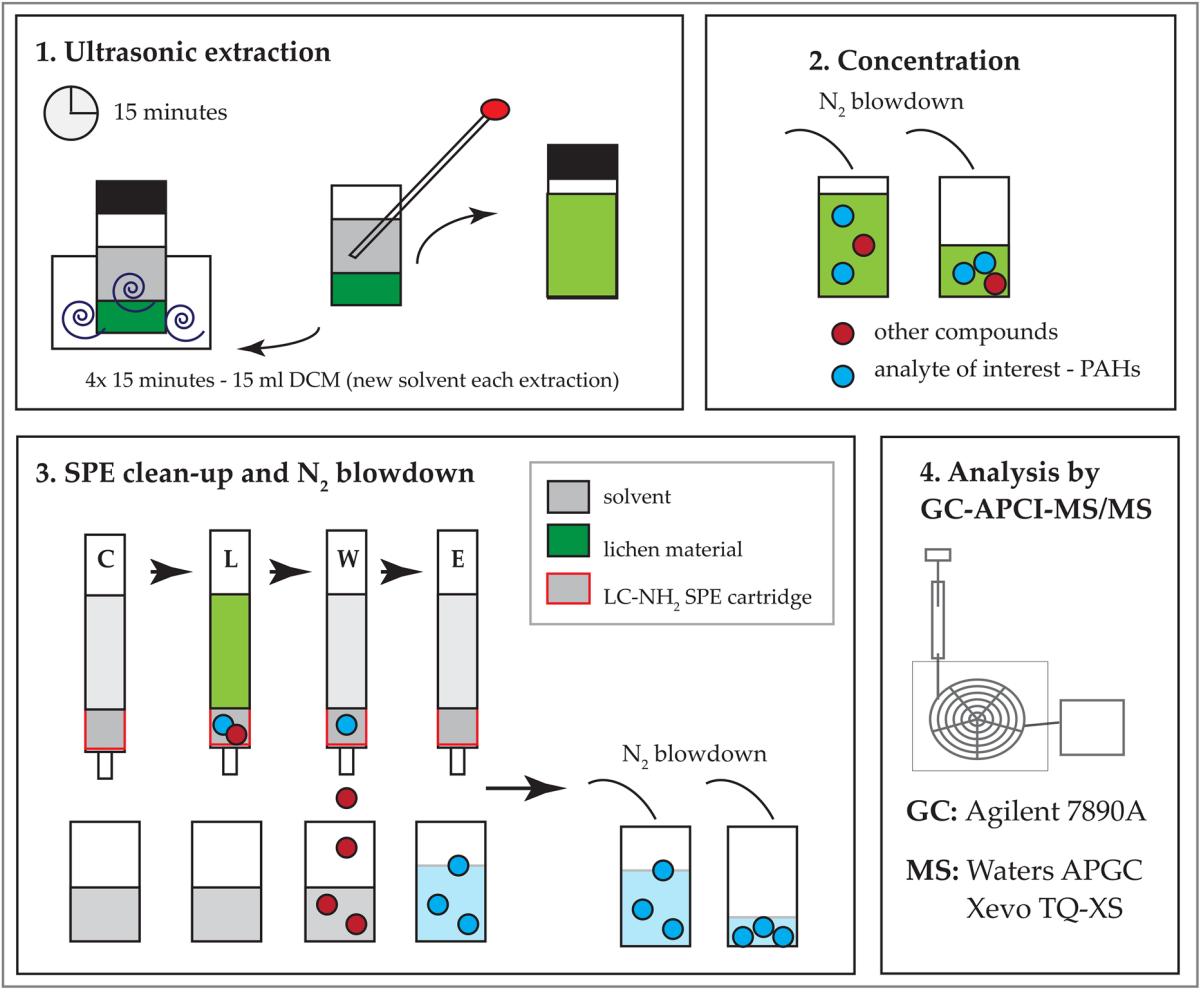
Lichen extraction procedure for PAH analysis (1) repeated ultrasonic-assisted extraction (4 × 15 min, using 15 mL of DCM), (2) pre-concentration to 3 mL, (3) solid-phase extraction (SPE, C condition, L load, W wash and E elution) using LC-NH_2_-SPE cartridges and concentration to 100 µl prior to (4) analysis by GC-APCI-TQ-MS/MS

Figure 2 illustrates the different steps undertaken for organic extraction of lichen material. Ultrasonic extraction (at 20 °C) was applied to lichen samples, with four sequential extractions of 15 min using 15 mL of DCM (Domeño et al., 2006; Käffer et al., 2012). After extraction, samples were centrifuged at 4000 rpm for 10 min to separate components. Supernatant was filtered through Whatman™ 540 filter paper (Fernàndez et al., 2011; Vitali et al., 2019) into new pre-cleaned glass vials via glass Pasteur pipettes (fired at 400 °C for 3 h and DCM rinsed prior to use). A total of 60 mL (4 × 15 mL of DCM) extraction solvent was blown down to 3 ml under a constant stream of pure nitrogen gas (N_2_) and subsequently cleaned by solid phase extraction (SPE).

SPE tubes (Supelco LC-NH2, 500 mg, 6 mL; Sigma-Aldrich, UK) were topped with 0.05 g anhydrous sodium sulphate and 0.05 g of Florisil® (100–200 mesh; Sigma-Aldrich/Merck, Germany) (Blasco et al., 2007, 2008, 2011; Concha-Graña et al., 2015; Domeño et al., 2006; Nascimbene et al., 2014). SPE conditioning comprised of 3 mL hexane and 6 mL DCM. Lichen extraction solution was added to the tube (flow rate: 1–2 drops min^−1^) and washed with 0.5 mL n-hexane. PAHs were eluted using 3 mL of n-hexane – DCM (3:1, v/v) (Domeño et al., 2006; Käffer et al., 2012). Toluene (20 µl; CHROMASOLV™ for pesticide residue analysis, Honeywell Riedel–de Haen™) was added to the sample, to avoid dryness and subsequently loss of more volatile compounds (i.e. naphthalene), and extracts were blown down under nitrogen to 100 µL prior to analysis.

The 16 EPA PAHs in lichen extracts were determined and quantified by gas chromatography atmospheric pressure chemical ionisation–tandem quadrupole–mass spectrometry (GC-APCI-MS/MS; GC: Agilent 7890A, MS: Waters APGC Xevo TQ-XS) at Waters Corporation (Wilmslow, UK)). The GC was equipped with a Rxi-5Sil-MS (30 m × 0.25 mm × 0.25 µm; Restek, UK) capillary column; helium was used as a carrier gas (constant gas flow at 2.0 mL min^−1^) and 1 µl was injected in pulsed-spitless mode (32 psi) into a multimode inlet (MMI) set at 310 °C. Analytical conditions of GC-APCI-MS/MS (adjusted from EPA, 1999) were set to an initial oven temperature of 50 °C (2 min hold), and temperatures were ramped at 20 °C min^−1^ to 150 °C (no hold) and 10 to 300 °C (hold for 5 min). Mass spectrometry was run in API + mode (APGC); the APCI corona pin was run at an electrical current of 2.0 µA; cone voltage was set at 5.0 V and N_2_ was used as cone gas, with a flow of 260 L h^−1^. The MS source and interface temperature was set at 150 °C and 280 °C, respectively.

The 16 EPA PAHs and deuterated PAH standards were identified by ‘Multiple Reaction Monitoring’ (MRM) using different collision energies (including qualifier and quantifier ions) as presented in Table S4.

### Quality assurance and quality control (QA/QC) – ICP-OES and ICP-MS

A six-point calibration was used for ICP-OES (Table S5), made from ESSLAB-910B (5% v/v HNO_3_) containing elements of interest. Signal-drift monitors were made from the same analytical standard, containing 2 µg mL^−1^ (Al, Ca, Fe, K, Na), 1 µg mL^−1^ (Cu, Mg, P, S) and 0.4 µg mL^−1^ (Cd, Co, Cr_3_, Mn, Mo, Ni, Pb, Zn and As), used to check for instrumental sensitivity through the analysis and were run after every two acid digested samples (Salit & Turk, 1998). Because of low variability (< % RSD – relative standard deviation) for each analytical batch of repeated signal-drift measurements, no signal-drift correction was undertaken for ICP-OES analysis.

ICP-MS calibration standard (five-point calibration; Table S5) and signal-drift monitor solutions were made up from Agilent Technologies multi-elemental standards: *multi-elemental standard-2A*, *multi-elemental standard-3*c and *multi-elemental standard-4b* (all Agilent Technologies, UK). Signal-drift solutions (containing 10 ng ml^−1^ of each element) for ICP-MS analysis were run after every sample (i.e. unknown and CRM acid digestion solution) and were used for drift correction for ICP-MS analysis, i.e. two signal-drift solutions bracketing an unknown sample solution (lichen sample or CRM) were averaged and related to the initially measured signal drift, resulting in a correction factor, which was applied to the sample (i.e. measured between the two signal drift solutions).

Replicate procedural blank solutions (*N* = 20) and lichen reference material (CRM No. 482; *N* = 23) were included within each sample digestion batch (*N* = 4), the latter to facilitate assessment of accuracy and precision of the lichen metal dataset. Accuracy and precision for lichen CRM metal concentrations are displayed in Table S6, which were measured after every five acid digested samples (‘unknowns’) for ICP-OES and ICP-MS analysis. Procedural blanks were used to determine methodological lower limits of detection (LLDs) for analysed elements, calculated separately for each analytical batch as three times the standard deviation (SD) of metal concentrations in blank solutions. LLD ranges (minimum to maximum) for analysed elements are displayed in the supplementary material (Table S3).

### Quality assurance and quality control (QA/QC)–GC–MS

Linearity of calibration standards, peak recognition, integration and PAH concentrations in lichen samples were determined by ‘Waters MassLynx V4.2’ software. Instrumental linear range was set between 0.1, 1, 10, 100 and 1000 pg µL^−1^. Calibration showed *R*^2^ > 0.98 for all compounds. A mid-range standard solution (10 pg µL^−1^) was re-measured to ensure instrumental performance, with a coefficient of variation (%CV) of 16 EPA PAHs ranging between 0.4% (chrysene) and 14% (benzo[b]fluoranthene) indicating the sufficiency of analytical results.

Calculation of PAH concentrations in lichen samples was undertaken in relation to deuterated standards: *phenanthrene d-10* for naphthalene, acenaphthylene, acenaphthene, fluorene, phenanthrene and anthracene; *chrysene d-12* for fluoranthene, pyrene, benz[a]anthracene, chrysene and *dibenzo[a,h]anthracene d-14* for benzo[b]- and benzo[k]fluoranthene, dibenzo[a,h]anthracene, indeno[1,2,3-cd]pyrene and benzo[ghi]perylene.

Analytical recovery rates (expressed as Rec%) for PAHs were analysed by spiking lichen material (*N* = 4) with certified reference material (CRM) ‘TCL PAH Mix’ (benzene:dichloromethane, Sigma-Aldrich, UK) containing 16 EPA PAHs at 2 ng µl^−1^ and deuterated PAH standards. PAH concentrations obtained for un-spiked samples were subtracted from spiked lichen material and used for calculation of spike recoveries following (Domeño et al., 2006). Recovery rates were found between 58% for naphthalene and 154% for benzo[a]pyrene (Table S7). Low recovery rates for low-molecular PAH naphthalene may be related to its high volatility (Song et al., 2002). In contrast, lichens were not washed prior to processing, extraction and analysis, suggesting potential incorporation of deposited material (i.e. PAH-containing particulates) into the procedure, explaining higher recoveries (> 120%) for benzo[b]fluoranthene and benzo[a]pyrene. However, correction for recovery rate on recorded lichen PAH concentrations was not undertaken, due to focus of this study on spatial variability of PAHs and potential fingerprinting of sources across Manchester.

A procedural blank, handled in the exact same way as lichen extraction samples (extraction and SPE clean-up), was used for blank subtraction of individual PAH concentrations, prior to calculation of lichen PAH concentrations (in ng g^−1^). Laboratory blanks (*N* = 4) were used to assess cross-contamination during GC-APCI-MS/MS analysis. Limits of detection (LOD) and limits of quantification (LOQ) were determined as three times (for LOD) and ten times (for LOQ) the noise in the chromatogram close to the compound of interest (Kodnik et al., 2015). LOD and LOQ for each PAH is displayed in Table S7.

### Statistical data analysis and visualisation

Statistical tools for data analysis and visualisation was undertaken using commercial software: Origin 2019 (OriginLab, 2018) and open-source software jamovi (The jamovi project, 2020) and R Studio (version 1.4.1103) with data visualisation package ‘ggplot2’ (RStudio Team, 2021; Wickham, 2016). Normality of lichen datasets for metal and PAH concentrations (by rings and individual) in lichens was done by Shapiro–Wilk test, due to higher statistical power regardless of sample size (Razali & Wah, 2011). Subsequent statistical analysis for metal and PAH concentrations were informed by outcomes of the ‘normal distribution’ test, for instance, correlation statistics were expressed as Pearson’s* r* (normally distributed data) or Spearman *ρ* (non-normally distributed data), whereas paired/unpaired *t* test statistics and non-parametric test statistics were used for non-parametric group comparison.

Comparison of sampling sites where both lichen species were obtained (*N* = 15) for metal concentrations was undertaken using Wilcoxon test statistics (non-parametric test) to investigate potential differences in uptake abilities and implications for lichen biomonitoring studies.

Urban factors to evaluate spatial variability and dispersion of atmospheric pollutants included traffic count data, distance to major roads (A-, B-roads and motorway; UK Department of Transport, 2012), proximal building heights (as mean building height of surrounding buildings). Justification and grouping of these publicly available datasets are described in Fig. S1. For instance, traffic counts statistics (annual average daily traffic flow, AADF), available for major roads (DfT, 2017a), was used to inform potential traffic emissions at sampling sites.

Due to potential of high PAH emissions from vehicle emission across Manchester, spatial variability was assessed in comparison to distance to road and traffic counts at the sampling location. Distance to road was classified based on reported decline of pollutant concentrations (i.e. NO_x_) within the first 200 m (Cape et al., 2004; Davies et al., 2007; Watmough et al., 2014). Here, distances were divided into two groups < 100 m and > 100 m, because all sampling sites were within 200 m of a major road and to assess PAH concentrations by potentially varying traffic influences, e.g. elevated lichen PAHs at highly trafficked roadside locations. Traffic counts (AADF) were sub-divided into < 20.000 and > 20.000 vehicles, and no sub-classification by vehicle types was undertaken, as majority of PAH emissions are related to ‘vehicular emissions’ covering diesel and gasoline-powered vehicles (Ravindra et al., 2008).

### Airborne metal source apportionment and human health risk assessment for potentially harmful elements (PHEs)

To investigate similarities within the lichen metal dataset, cluster analysis was used following the methodology described by (Doǧrul Demiray et al., 2012): metal concentrations were standardised by subtracting the mean concentration from each sample (individually for each metal) and dividing by the standard deviation (*z* score) before clustering. Standardisation was used to compensate for different magnitudes in elemental concentration of lichen samples (Doǧrul Demiray et al., 2012). Agglomerative hierarchical clustering, with complete linkage (furthest neighbour) and squared Euclidian distance, were used to produce dendrograms of similarity/dissimilarities (Doǧrul Demiray et al., 2012), which was subsequently used to aid potential source apportionment of recorded lichen metal concentrations.

The air pollution level for airborne metal concentrations across Manchester was determined by calculating the pollution index factor (PIF; Bozkurt, 2017), following Eq. 1.

1 $$PIF={C}_{s}/{C}_{b}$$

*C*_*s*_ is the elemental concentration (As, Cd, Cr, Mn, Ni and Pb) in the lichen *X. parietina*, due to more samples obtained for this lichen species, whereas *C*_*b*_ is the background concentration for the element, obtained from a controlled area or lowest concentration value detected for each element (Boamponsem et al., 2010; Bozkurt, 2017; Salo et al., 2012). Because *X. parietina* samples were also sampled from a rural environment (*N* = 12; Table S8, data not further discussed), the average elemental concentration for target element of the rural samples was used for *C*_*b*_.

Subsequently, the pollution load index PLI of lichen samples for each sampling location is calculated following Eqs. 2 and 3:

2 $${{\mathrm{PLI}}}_{{\mathrm{SamplingSite}}}={({{\mathrm{PIF}}}_{{\mathrm{element}}1}\times {{\mathrm{PIF}}}_{{\mathrm{element}}2}\dots \times {{\mathrm{PIF}}}_{{\mathrm{elementN}}}))}^{{~}^{1}\!\left/ \!{~}_{n}\right.}$$

3 $${{\mathrm{PLI}}}_{{\mathrm{All}}}={({{\mathrm{PIF}}}_{{\mathrm{SamplingSite}}1}\times {{\mathrm{PIF}}}_{{\mathrm{SamplingSite}}2}\dots \times {{\mathrm{PIF}}}_{{\mathrm{samplingSiteN}}})}^{{~}^{1}\!\left/ \!{~}_{n}\right.}$$

To assess the degree and intensity of pollution, PLIs were calculated for urban morphology types (UMTs; Fig. 1) with PIF < 1.2: unpolluted, 1.2–2.0: lightly polluted, 2.0–3.0: medium polluted and > 3.0: heavily polluted (Boamponsem et al., 2010; Bozkurt, 2017). To better compare PLIs, UMTs were combined and categorised into broader ‘classes’, consisting of town centre (including UMTs: town centre, retail and offices: *N* = 23), residential (including UMTs: high and medium density residential; *N* = 33), industrial (including UMTs: manufacturing, disused and derelict land, schools; *N* = 17), green space (including UMTs: formal and informal open space; *N* = 7) and major road (*N* = 4).

Potential human health effects of exposure to (non-)carcinogenic airborne pollutants were evaluated according to the U.S. EPA and the Netherlands’ National Institute of Public Health and Environmental Protection, using the three major pathways (ingestion, inhalation and dermal contact) of human PHE (As, Cd, Cr, Ni, Pb, Cu and Zn) exposure, which were calculated as the average daily dose (ADD) as shown in Eqs. (4), (5) and (6) (EPA, 1989, 1996; Khodadadi et al., 2023; van den Berg, 1994) using the variables in Table 1. In this study, manganese (Mn) was included as PHE, due to its potential neurotoxicological long-term effects on humans (Šaric & Lucchini, 2017). The hazard quotient (HQ) and hazard index (HI) were calculated using ADD_ing_, ADD_inh_ or ADD_derm_ using Eqs. (7) and (8). To calculate HQ, the reference dosage (RfD) [mg/kg/day] as an estimate of the maximum daily absorption permitted during human life, used to divide the average daily dose (ADD) (Khodadadi et al., 2023; Man et al., 2010). RfDs for ingestion, inhalation and dermal contact for As, Cd, Cr, Mn, Ni, Pb, Cu and Zn are displayed in Table S9 (Goudarzi et al., 2021; Kamunda et al., 2016; Khodadadi et al., 2023)

**Table 1:** Explanation of variables used in calculation for human health risk assessment of potentially toxic elements (PTEs) in Eq. (1) to Eq. (9), as described in: Khodadadi et al., 2023; Li et al., 2020; Pan et al., 2019; Rabin et al., 2023; Sun & Chen, 2018 and references therein

Abbrev	Name	Value		Unit
		Child	Adult	
ADD	Average daily dose	Calculated		mg/kg/day
C	(Average) elemental concentration in lichen samples (*X. parietina*)	Measured		mg/kg
Ring	Rate of ingestion	200	100	mg
Rinh	Rate of inhalation	20	7.6	m^3^
EF	Exposure frequency	350		day/year
ED	Exposure duration	6	24	years
BW	Bodyweight	15	70	kg
AT	Average time	ED × 365 for non-carcinogenic elements; 25,550 days (lifetime) for carcinogenic elements		day
PEF	Particle emission factor	1.36 × 10^9^		m^3^/kg
SA	Exposed skin surface area	2800	5700	cm^2^
SL	Skin’s airiness factor	0.2	0.07	mg/cm^2^
ABS	Skin absorption coefficient	0.001 for all elements; 0.03 for arsenic		unitless
HQ	Hazard quotient	Calculated		unitless
HI	Hazard index	Calculated		unitless
RfD	Reference dose of the individual PTE	Table S9		mg/kg/day
CCR	Cumulative carcinogenic risk	Calculated		unitless
CR	Carcinogenic risk	Calculated		unitless
SF	Carcinogenicity slope factor	Table S9		per mg/kg/day

4 $${{\mathrm{ADD}}}_{{\mathrm{ing}}} \;(\mathrm{mg}\;{{\mathrm{kg}}}^{-1} {{\mathrm{day}}}^{-1})=\mathrm{C }\;(\mathrm{mg }\;{{\mathrm{kg}}}^{-1})\times \frac{{\mathrm{Ring}}\times {\mathrm{EF}}\times {\mathrm{ED}}}{{\mathrm{BW}}\times {\mathrm{AT}}}\times {10}^{-6}$$

5 $${\mathrm{ADD}}_{\mathrm{inh}}\;(\mathrm{mg}\;\mathrm{kg}^{-1}\mathrm{day}^{-1})=\mathrm C\;(\mathrm{mg}\;\mathrm{kg}^{-1})\times\frac{\mathrm{Rinh}\times\mathrm{EF}\times\mathrm{ED}}{\mathrm{PEF}\times\mathrm{BW}\times\mathrm{AT}}\times10^{-6}$$

6 $${\mathrm{ADD}}_{\mathrm{derm}}\;(\mathrm{mg}\;\mathrm{kg}^{-1}\mathrm{day}^{-1})=\mathrm C\;(\mathrm{mg}\;\mathrm{kg}^{-1})\times\frac{\mathrm{SA}\times\mathrm{SL}\times\mathrm{ABS}\times\mathrm{EF}\times\mathrm{ED}}{\mathrm{BW}\times\mathrm{AT}}\times10^{-6}$$

7 $${\mathrm{HQ}}=\frac{{\mathrm{ADDing}},\mathrm{ inh \;or \;derm}}{{\mathrm{RfDing}},\mathrm{ inh \;or \;derm}}$$

8 $${\mathrm{HI}}=\sum\nolimits_{i=1}^{n}HQ$$

HI, as the sum of the HQs, is the potential human health risk associated with all exposure pathways, where a value of > 1 indicates negative effects in human health (Khodadadi et al., 2023; Kong et al., 2011). The cumulative carcinogen risk (CCR) to human health can be calculated according to Eq. (9), using the ADD_ing/inh/dermal_ and the slope factor of PTEs to calculate the cancer risk (CR) for each exposure pathway (ingestion, inhalation and dermal; Table S9).

9 $${\mathrm{CCR}}=\sum {\mathrm{CR}}={\mathrm{CRing}}+{\mathrm{CRinh}}+{\mathrm{CRdermal}}$$

### PAH diagnostic ratios and human health risk assessment

Atmospheric PAHs originate from numerous sources, both petrogenic (e.g. crude oil, gasoline, asphalt and coal) and pyrogenic (e.g. combustion engines, fires and furnaces; Mauro & Roush, 2008). For instance, pyrogenic PAHs are formed during burning of organic substances at high temperatures (‘pyrolysis’), consisting of larger rings, compared to petrogenic PAHs that are generated at lower temperatures (Hussain et al., 2018). Differentiation between PAH sources, i.e. coal-, wood- or oil-based can be undertaken using chemical fingerprinting, i.e. PAH diagnostic ratios, which has been applied in lichen biomonitoring studies (Augusto et al., 2016; Blasco et al., 2006, 2008; Fernàndez et al., 2011; Shukla & Upreti, 2009; Shukla et al., 2012).

Diagnostic ratios used are displayed in Table 2 and were used to potentially identify primary PAH sources across Manchester. Additionally, combustion PAHs (PAH_comb_) against total PAHs (PAH_total_) was used as indicator, together with other ratios, to confirm the origin (Augusto et al., 2016; Hwang et al., 2003). PAH_comb_ includes fluoranthene (FLT), pyrene (PYR), benzo[a]anthracene (BaA), chrysene (CHRY), benzo[b]Fluoranthene (BbF), benzo[k]fluoranthene (BkF), benzo[a]pyrene (BaP), indeno[1,2,3-cd]pyrene (IcdP) and benzo[ghi]perylene (BghiP) (Hwang et al., 2003). To provide safer interpretation, PAH diagnostic ratios were further cross-plotted (Yunker et al., 2002) and analysed in relation to road distances and traffic count statistics (as described in “Quality assurance and quality control (QA/QC)–GC–MS” section).

**Table 2:** PAH diagnostic ratios applied in lichen biomonitoring studies and potential sources. *ANT* anthracene, *PHE* phenanthrene, *NAP* naphthalene, *FLT* fluoranthene, *PYR* pyrene, *BaA* benzo[a]anthracene, *CHRY* chrysene, *combPAH* combustion PAH

PAH ratios	Source
ANT/(ANT + PHE)	< 0.10—petroleum > 0.10—combustion
NAP/PHE	High ratios—local sources < 1—petroleum
FLT/(FLT + PYR)	> 0.5—grass, wood, coal combustion 0.4–0.5—gasoline, diesel and crude oil combustion (car and diesel trucks) < 0.4—petroleum
FLT/PYR	< 1.0—vehicular emissions and industrial and domestic sources (petroleum) > 1—combustion
PHE/ANT	< 10—vehicular emissions and industrial and domestic sources > 10—petrogenic sources (petroleum)
BaA/(BaA/CHRY)	> 0.35—pyrogenic
CombPAH/PAH	> 0.7—combustion sources

(Augusto et al., 2016; Blasco et al., 2006, 2008, 2011; Fernàndez et al., 2011; Satya et al., 2012; Shukla et al., 2012; Shukla & Upreti, 2009)

Evaluation of toxicity and assessment of human exposure to PAHs followed the methodology outlined by the U.S. EPA (EPA, 1993), using PAH-individual carcinogenic potencies, so-called ‘Toxic Equivalence Factors (TEFs)’ (Table S10), expressed as equivalent concentrations of benzo[a]pyrene Eq. (10) (Augusto et al., 2013; EPA, 1993; Nisbet & LaGoy, 1992).

10 $${{\mathrm{BaP}}}_{{\mathrm{eq}}}= \sum\nolimits_{i=1}^{16}({C}_{i} \times {{\mathrm{TEF}}}_{i})$$

With *Ci* being the concentration of the PAH and TEF_*i*_ as the toxic equivalence factor for the specific PAH (Table S10; Nisbet & LaGoy, 1992). The total carcinogenic potency was calculated for each sampling site using median PAH concentrations (Sarigiannis et al., 2015) in *X. parietina* (*N* = 20).

Potential health risk assessment of PAHs via inhalation exposure was calculated using the incremental lifetime cancer risk (ILCR) following U.S. EPA guidance (EPA, 2005) as shown in Eq. (11).

11 $${\mathrm{ILCR}}={{\mathrm{UR}}}_{{\mathrm{BaP}}}\times {{\mathrm{BaP}}}_{{\mathrm{eq}}}$$

With UR_BaP_ is the unit cancer risk via inhalation exposure to one unit of benzo[a]pyrene (1 ng m^−3^) over 70 years (median human lifespan), which is set 8.7 × 10^−5^ ng m^−3^ by the WHO based on an epidemiologic study (WHO, 2000; Yang et al., 2019). BaPeq was calculated as shown in Eq. (10).

## Results and discussion

### Temporal variability of metal concentrations and implications for lichen PAH concentrations

To evaluate a potential temporal bias superimposed on lichen metal concentrations, a sub-set of sampling sites (*N* = 17, sampled for *X. parietina*) was revisited in 2018, and metal concentrations were compared to samples from the initial sampling period in 2016/2017 (Fig. 1a). A general increase of metal concentrations in *X. parietina* was observed for Cd, Cr, Mn and Ni, whereas Pb was recorded at lower concentrations in 2018 (Fig. S2). For target metals in this study, only Cr and Pb showed statistically different (*p* < 0.05) differences between sampling periods.

Accumulation patterns in lichens depend on length of exposure and/or lichen age (Coccaro et al., 2000; Garty, 2001). Lichen transplantation studies reported that most lichen species respond to changing atmospheric metal concentrations within a few months (Bačkor & Loppi, 2009). For instance, lichen biomonitoring studies in rural, urban and industrial sites in Italy and New Zealand reported changes in lichen metal concentrations between 6 and 15 months (Kularatne & De Freitas, 2013; Loppi et al., 2004; Paoli et al., 2018a, 2018b). Seasonal changes of lichen metal concentrations have also been recorded, with precipitation having a ‘wash-off’ effect of metal-containing particulates, or contribution of elements from rainfall (Bačkor & Loppi, 2009; Corapi et al., 2014; Knops et al., 1991; Vannini et al., 2017). Comparably, Kularatne & De Freitas (2013) reported higher on-thallus accumulation (dry deposition) during summer months, whereas a direct impact of precipitation on accumulation and release of metals from the lichen surface was also recorded. Moreover, when wet, lichens are metabolically active, and temporal variation could be related to uptake during wet periods (i.e. winter months; Bačkor & Loppi, 2009; Nash & Gries, 1995). Nonetheless, metal concentrations in lichen thalli vary according to the amount of pollutants and connected biological stress, and in turn, altering elemental uptake. For *X. parietina*, different accumulation abilities in vegetative parts (i.e. thallus and apothecia) were reported (Rola & Osyczka, 2019). Moreover, Paoli et al., (2018a, 2018b) reported that Pb is preferentially stored at extracellular level (i.e. cell wall binding sites) in *X. parietin*a, and temporal variability could be explained by a wash-off effect from the lichen surface (Bačkor & Loppi, 2009; Garty, 2001; Hauck & Huneck, 2007).

In this study, young lichens were sampled from twigs and branches to assess recent air quality, and samples were not washed prior to grinding and acid digestion to include entrapped/deposited particulates and obtain an ‘overall’ metal concentration, rather than bioconcentrated portions (Forbes et al., 2015). Although a temporal bias for Cr and Pb has been recorded, assessment of spatial variability was further undertaken, because both pose a significant risk on human health (Kampa & Castanas, 2008). For Manchester in particular, Cr is closely linked to vehicular emissions, whereas Pb was recorded at concentrations up to 645 µg g^−1^ in road dust samples within the city centre (Robertson et al., 2003). However, it is suggested that sampling should be undertaken during short periods, to minimise potential temporal bias. Further, careful consideration of the complex physiochemical processes of metal accumulation in lichens is needed when applying a biomonitoring study.

As temporal variability was recorded for airborne metal concentrations, it is strongly suggested to consider temporal variability of atmospheric PAH concentrations in lichen biomonitoring studies. For instance, varying PAH concentrations in lichens and seasonal variations of atmospheric PAHs, i.e. high in winter (due to low temperatures) and low in summer (due to high evaporation and volatilisation) have been reported (Augusto et al., 2016, 2013; Garrido et al., 2014; Kodnik et al., 2015; Shukla et al., 2012). Accumulation of PAHs in lichens is dependent on wet or dry deposition and the relative solubility of PAHs, different molecular weights of compounds and resistance imposed by the thallus morphology, structure and roughness, subsequently intercepting and retaining airborne particles (Augusto et al., 2015; Bergamaschi et al., 2007; Blasco et al., 2011; Shukla et al., 2014). Augusto et al. (2013) highlighted the drawback of lichen-derived PAH concentrations and their ‘translation’ into atmospheric concentrations. They used a combined approach, using *Parmotrema hypoleucinum* and particulate-phase samplers (active air sampling) and reported seasonal variation in lichens and ambient PAH concentrations that followed a similar trend. Comparable results were reported combining a lichen biomonitoring approach with passive sampling devices (e.g. containing polyurethane foam disks) (Domínguez-Morueco et al., 2015; Loppi et al., 2015). Hence, a relatively short sampling period to assess PAH concentrations in lichens, to minimise temporal bias, is suggested.

In this study, lichens were obtained during dry days (i.e. no forecasted precipitation for a day) between May and October 2018, suggesting potential impacts by meteorological variables (i.e. precipitation and temperature) and thus, temporal bias that could not be fully accounted for, requiring careful interpretation of the lichen PAH dataset. However, lichen samples were quickly transported (in paper bags) to the laboratory, processed (i.e. scraped off the bark and freeze-dried) and stored frozen until analysis. Hence, spatial variability of lichen PAH concentrations could be used to assess deteriorated air quality within the urban environment of Manchester.

### Spatial variability of metal concentrations in lichen samples—*X. parietina* and *Physcia* spp.

Spatial variability of metal concentrations was recorded for both lichen species, with generally higher concentrations in *X. parietina* and statistically significant differences (*p* < 0.05, As, Cd, Cr and Mn;* p* < 0.01, Pb) between lichen species (Table S11).

**Fig. 3:**
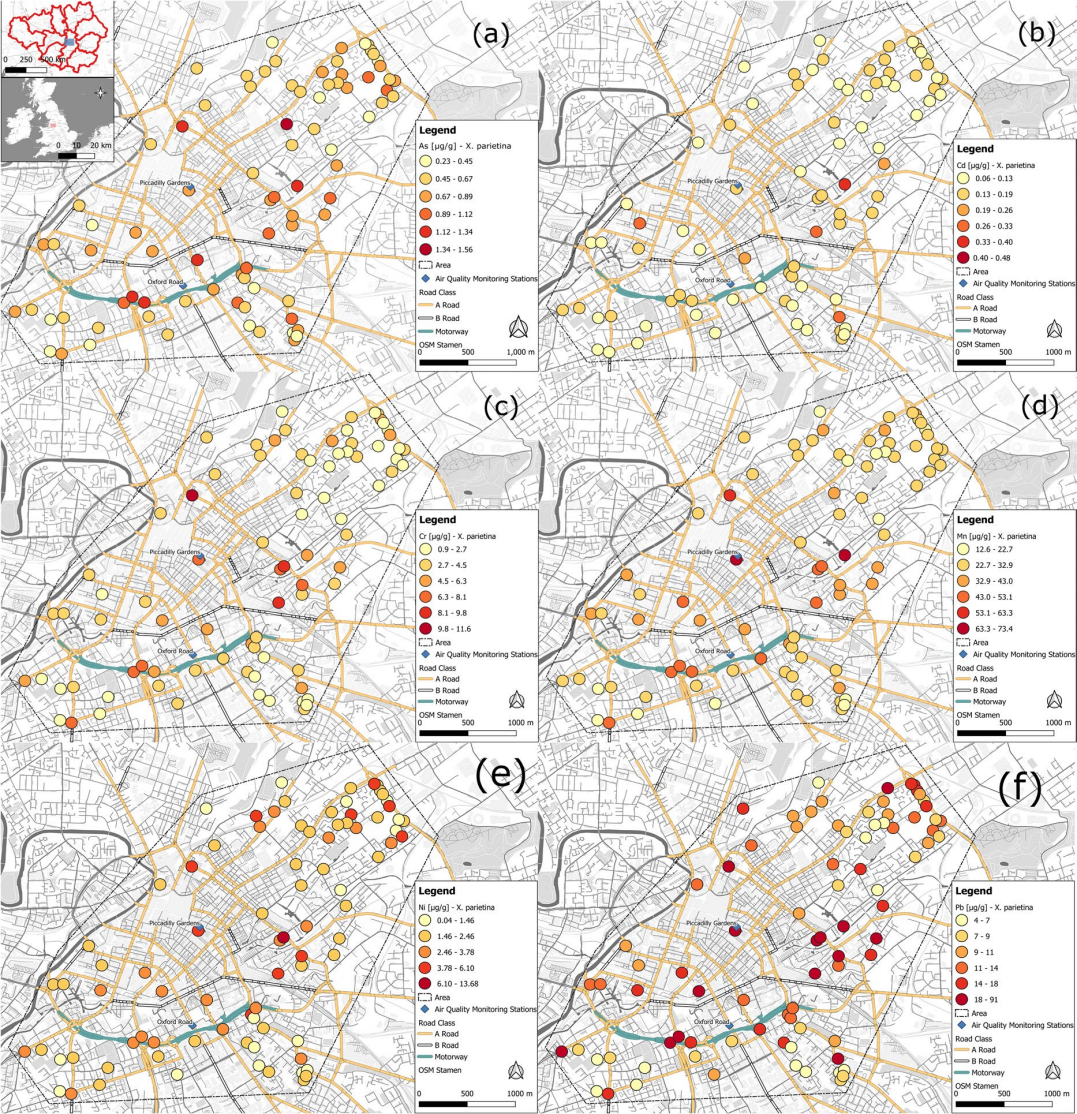
Metal concentrations (in µg g.^−1^) recorded for *X. parietina*, for (**a**) arsenic, (**b**) cadmium, (**c**) chromium, (**d**) manganese, (**e**) nickel and (**f**) lead, across Manchester city centre (colour-coded from low to high); displayed with automated air quality monitoring stations and major road network (A- and B-roads and motorway)

Arsenic in air is predominantly associated with particulate matter (Chung et al., 2014), and historically, the largest source of arsenic in the UK was linked to coal combustion, which has decreased by 98% (since 1990), whereas nowadays, burning of treated wood (61% in 2019) and iron and steel production (19% in 2019) are the major arsenic pollution sources (NAEI, 2019). Moreover, in the UK, naturally elevated levels of arsenic have been reported in soils (Ander et al., 2011; Cave et al., 2013). Hence, elevated arsenic concentrations in lichen samples along Manchester’s major road network (Fig. 3a) suggests particulate-/soil-deposited material on the lichen surface. Comparably, Ni is naturally found in soils, whereas anthropogenic sources are linked to industrial manufacturing (e.g. steel and electroplating), fossil fuel combustion and engine wear (ATSDR, 2005; EEA, 2015; Taylor, 2006). For instance, Parzych et al. (2016) and Kurnaz and Cobanoglu (2017) reported elevated Ni levels in lichens at urban roadside locations and sites impacted by industrial activities in Italy and Turkey, findings that are comparable to results presented for Manchester (Fig. 3e).

Anthropogenic sources of Cd are linked to fossil fuel combustion, iron, steel and non-ferrous metal production and waste incineration (ATSDR, 2012; Kurnaz & Cobanoglu, 2017), whereas Cr (and Mn) are linked to vehicular wear (e.g. brake linings and lubricant oil; (Charron et al., 2019; Pulles et al., 2012; Taylor, 2006) and exhaust fumes (Kurnaz & Cobanoglu, 2017; Taylor, 2006). Lichen Cd and Cr concentrations for Manchester (Fig. 3b and c) were comparable to results reported at highly trafficked and congested areas of Istanbul (Turkey; Kurnaz & Cobanoglu, 2017) and across Rieti’s urban area (Italy; Owczarek et al., 2001) that identified vehicular sources (e.g. tyre and vehicle wear and alloys; Taylor, 2006) as primary pollution source. However, for Manchester highest Cd was recorded further away from a major road (Fig. 3b). Cd can be associated to particulates allowing long distance travel (i.e. from traffic sources), suggesting a potential particulate-bound Cd influence (Adamo et al., 2011; Vingiani et al., 2015). In contrast, Vingiani et al. (2015) reported lower Cr concentrations in deployed lichens (*P. furfuracea* and *Parmelia sulcata*) at heavy and moderately trafficked locations in London. Such differences could be linked to species-specific differences in sensitivity to airborne Cr and the applied study design, i.e. passive biomonitoring—this study and active deployment (Vingiani et al., 2015).

Mn in lichen thalli is directly proportional to atmospheric concentrations, with about 50% of airborne Mn in urban areas being of natural/geological background (Parzych et al., 2016). However, the highest Mn was along the major road network (Fig. 3d) and in proximity to Manchester’s main bus terminal (‘Piccadilly Gardens’) with approximately 3700 buses daily (DfT, 2017a) of which 20% fall into the most polluting EURO 2 and 3 emission standard (Cox & Goggins, 2018).

Historically, lead has been used as antiknock additive to petroleum fuel but was phased out in 2000. Due to its environmental persistence and severe human health impact, it is still a relevant environmental pollutant (Adamiec et al., 2016; Nagajyoti et al., 2010). Pb concentrations of 357 µg g^−1^ have been reported in road dust sediments within the Manchester’s city centre (Barrett et al., 2010; Robertson et al., 2003), and elevated lichen Pb could be linked to re-suspension of soil particles and dust, deposited onto the lichen surface.

Lichen metal concentrations were positively correlated with each other, e.g. Mn was positively correlated (*p* < 0.001; Table S12) with Ni (and Cr) and a decline of Mn concentrations with increasing distance to major roads suggesting vehicular and engine wear as primary source (Charron et al., 2019; Gehrig et al., 2007; Kurnaz & Cobanoglu, 2017; Taylor, 2006). Additionally, positive correlation of Pb (in *X. parietina*) with Mn (*r* = 0.79, *p* < 0.001) and Cd (*r* = 0.53, *p* < 0.001) also suggests local traffic-related sources as primary cause of pollution (Doǧrul Demiray et al., 2012).

**Fig. 4:**
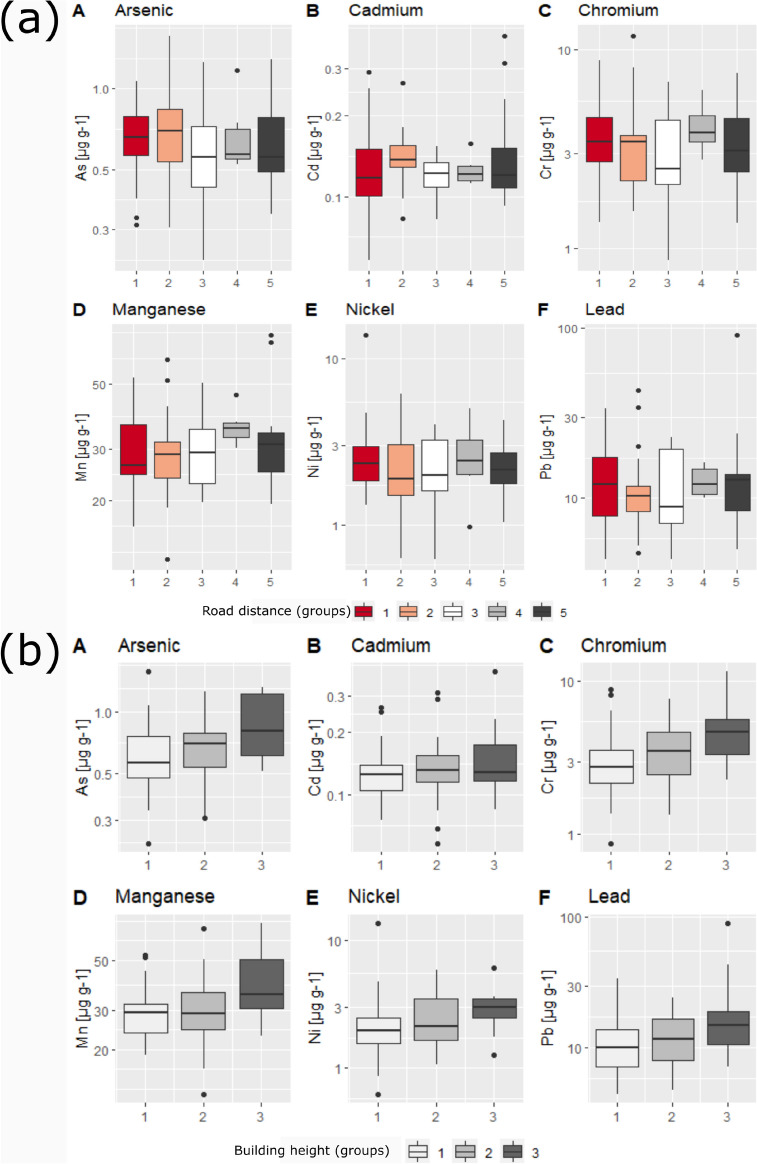

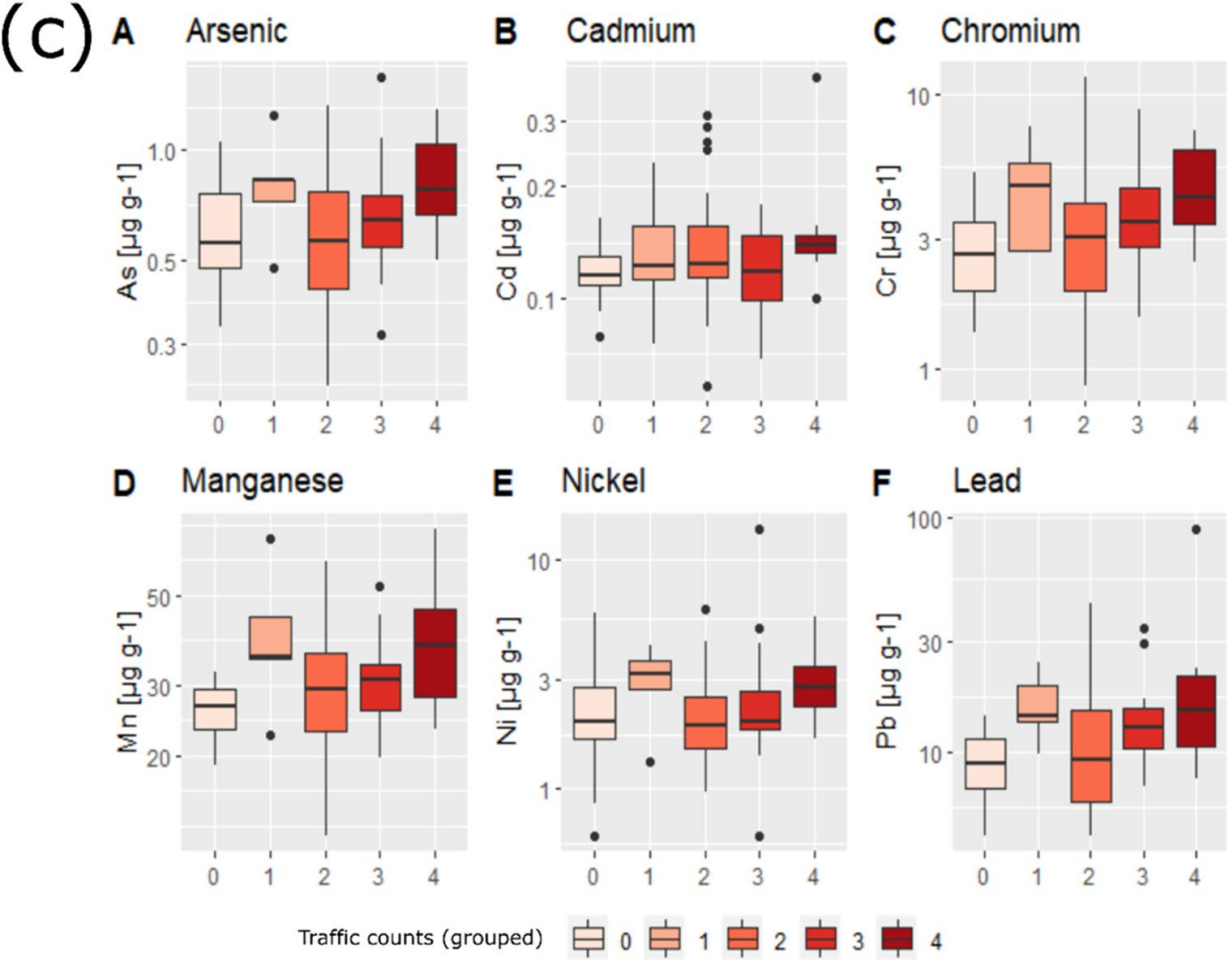
Lichen-derived (*X. parietina*) metal concentrations displayed as boxplots (25th to 75th percentile) for **A** As, **B** Cd, **C** Cr, **D** Mn, **E** Ni and **F** Pb by distances for grouped data. (**a**) Major road distance (1, < 25 m; 2, 25–50 m; 3, 50–100 m; 4, 100–200 m and 5, > 200 m). (**b**) Average surrounding building heights (1, < 10 m; 2, 10–20 m; 3, > 20 m). (**c**) Traffic count data (0, N/A no data available, 1, < 10.000; 2, 10.000–20.000; 3, 20.000–30.000; 4, > 30.000)

Statistical comparison of lichen metal data with (grouped) urban influencing factors showed no statistically significant differences of means for metal concentrations and distance to major road (Fig. 4a). However, Mn and Cr were significantly negative (Spearman ρ, *p* < 0.05) correlated with distance to major road in *X. parietina*. Comparably, Cr, Mn and Pb showed statistically significant differences (Kruskal–Wallis, *p* < 0.05) of means (Fig. 4c) and a significant (Spearman ρ, *p* < 0.05) positive correlation with traffic count data. For surrounding building heights (grouped), only Cr showed statistically significant differences (*p* < 0.05) in *X. parietina*. However, when applying a post-hoc Dwass-Steel-Critchlow-Flinger (DSCF) pairwise comparison of building height groups and lichen metals, group 3 (> 20 m) was found significantly (*p* < 0.05) different from group 1 (< 10 m) for As, Cr and Mn (Fig. 4b). Moreover, Cr, Ni and Pb in lichens showed statistically significant (*p* < 0.05) positive correlation for surrounding building heights, illustrating impeded air ventilation and increased metal concentrations at more densely built up locations.

Compact urban forms (of high density) affect wind flow patterns, resulting in pollutant accumulation and poor air quality, particularly in street canyons with high buildings tend to have heavier pollution levels, particularly at pedestrian level (Buccolieri et al., 2009; Fu et al., 2017; Shen et al., 2017). Longley et al. (2004) reported the complex influence between urban topography, wind (within and above a street canyon) and vertical turbulences by traffic on dispersion of pollutants in a street canyon of Manchester. Results presented suggest ‘canyoning’ effects across Manchester. Overall, spatial variability of lichen metal concentrations in Manchester suggests (local) vehicular emissions (including potential association with lichen surface deposited particulates) as primary sources of pollution. Concentrations of target metals (As, Cd, Cr, Mn, Ni and Pb) were found highest along the major road network and within the city centre area for both lichen species (*X. parietina* displayed in Fig. 3 –*Physcia* spp. data not shown; *N* = 17) showing deteriorated air quality by airborne metals in the centre of Manchester.

In this study, only limited datasets were used to incorporate potential urban layout effects on recorded metal concentrations. For instance, major roads and traffic counts (primarily available for major roads—A-, B-roads and motorways) were considered representative for potential high pollutants sources, due to limited data for minor and unclassified roads (UK Department of Transport, 2012, 2018). Additionally, it needs to be stated that evident species-specific differences suggest the use of a single lichen species, when comparing (urban) environments. For instance, chromium is detrimental to plant growth and development, and non-significant relationship for *Physcia* spp. could be related to toxic effects of Cr (Dzubaj et al., 2008; Kováčik et al., 2018; Sanità Di Toppi et al., 2004) and/or because only a small number (*N* = 17; data not shown/discussed) has been analysed.

### Total and individual PAH concentrations in X. parietina samples across Manchester’s city centre in comparison to other urban lichen biomonitoring studies

**Fig. 5:**
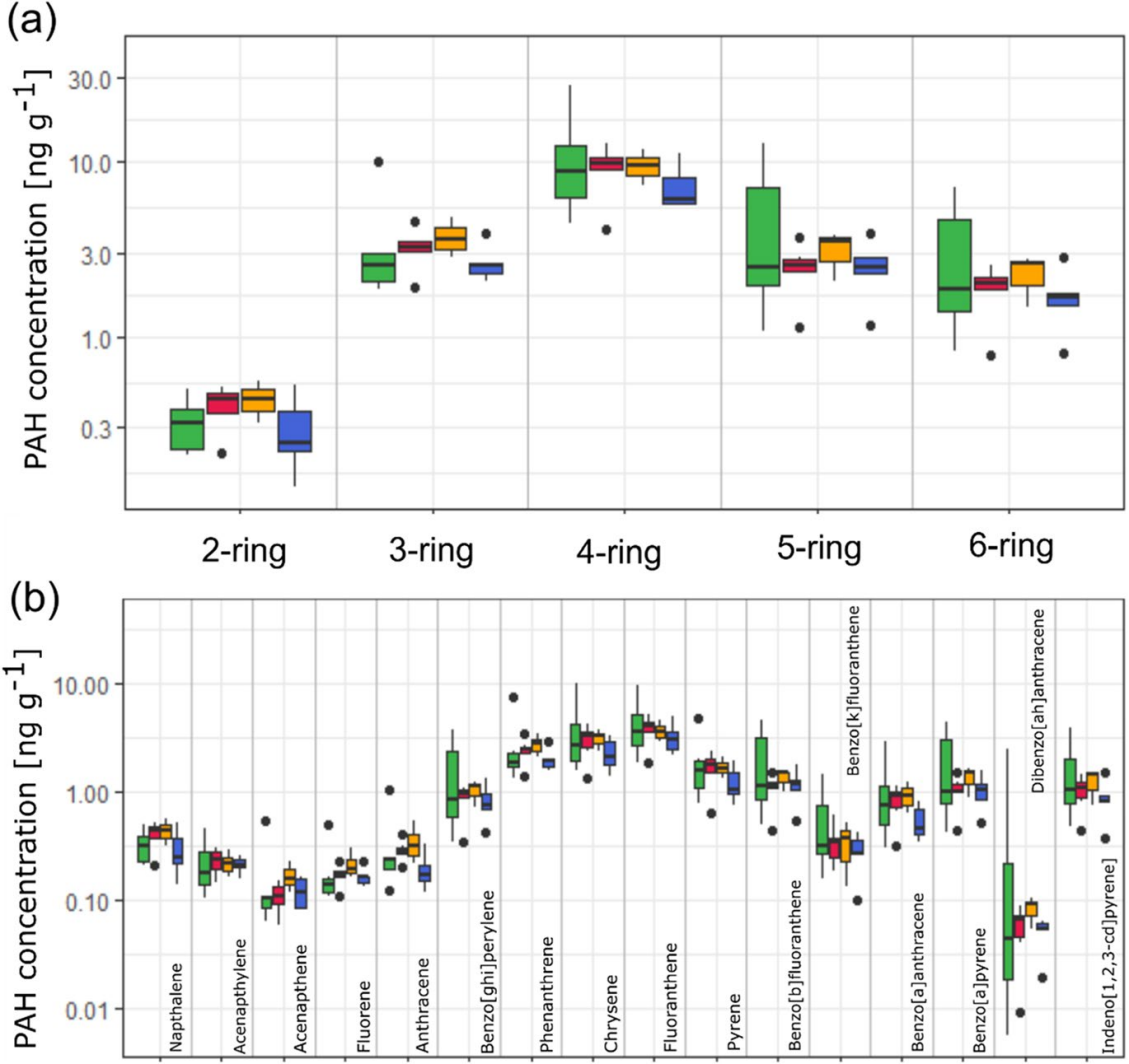
Individual PAH concentrations [in ng g^−1^] in *X. parietina* (**a**) and total lichen PAH concentrations by ring structure (**b**); colour-coded by land-use type (as shown in Fig. 1): green = green space (GS); red = major road (MR); orange = major road/residential (MR/RES) and blue = residential (RES)

The use of PAH concentrations, summarised by rings, is considered indicative for pollutant sources, whereas individual concentrations provide information on the gradient of pollution (Augusto et al., 2009). Figure 5a and b illustrate total (∑PAH) and individual PAH concentrations (colour-coded by location groups; Fig. 1b, Table S13) recorded in *X. parietina*.

Total lichen PAH profiles by land-use classes were GS > MR > RES > MR/RES, with a predominance of 4-ring PAHs, at a total concentration of 189.82 ng g^−1^ and contributing between 38 and 58% of the total PAH profile. Comparably, 3-, 5- and 6-ring PAHs contributed between 6 and 23%, 11 and 13% and 8 and 22% the total PAH profile, respectively, whereas 2- PAHs showed only minor contribution (1–3%).

Two- and three ring PAHs are present in the vapour phase of the atmosphere, whereas 4-ring PAHs can be present in both, gas and particulate phase, whereas 5- and 6-ring PAHs are associated with particulates (Augusto et al., 2010, 2015). In general, urban areas are dominated by 4-ring PAHs, whereas industrial areas are dominated by 5- and 6-ring PAHs (Augusto et al., 2009; Guidotti et al., 2003), with increases in 4-, 5- and 6-ring lichen PAHs indicating local PAH sources (Vingiani et al., 2015).

Findings for Manchester are in accordance with findings by Augusto et al. (2010), who reported a predominance of 4-ring PAHs in *X. parietina* samples of the Sines region, Portugal. Analogous, Owczarek et al. (2001) reported elevated 4-ring PAHs in *Physcia adscendens* samples from Rieti’s (Italy) urban area. In contrast, Blasco et al., 2006, (2008) reported 3-ring PAHs in *Evernia prunastri* and *Parmelia sulcata* along a highly trafficked national road in the Aragon Valley, Spain. Slezakova et al. (2010) reported higher concentrations of 5- and 6-ring PAHs in particulate matter (PM) at traffic influenced sites. Elevated concentrations of high-molecular PAHs across Manchester suggest particulates deposited on the lichen surface (i.e. from diesel and gasoline cars; Shukla et al., 2013). Comparable results have been reported for in lichens from London (UK) suggesting a similar PAH profile across urban environments in the UK. Nonetheless, recorded PAH concentrations suggest a complex mixture of PAHs across Manchester. Notably, elevated PAH concentrations, including more toxic PAHs, were recorded within green spaces that are potentially related to dispersion influences caused by the urban surrounding (e.g. surrounding building structures) and resuspension of contaminated soils deposited on the lichen surface. In contrast, 2- and 3-ring PAHs (more volatile) in lichens are reportedly associated with vehicular emission and can also be transported further away from their source (Blasco et al., 2008; Vingiani et al., 2015). Nonetheless, differences could be related to lichen species-specific (uptake) abilities or varying sources in the different environments.

Individual PAH concentration ranges in *X. parietina* were highest for pyrene (4-ring), fluoranthene (4-ring) and phenanthrene (3-ring), ranging between 1.84 and 9.63 ng g^−1^, 1.32–9.90 ng g^−1^ and 1.32–7.55 ng g^−1^, respectively. Interestingly, the most toxic PAH, benzo[a]pyrene, was recorded highest at green spaces sites (ID 4 and ID 14; Fig. S4; Fig. 5b; > 3 ng g^−1^), with concentration ranges between 0.42 and 4.33 ng g^−1^ (Fig. 5; Table S13). Benzo[a]pyrene is emitted by vehicles with and without catalytic converter and domestic coal and wood-burning and industrial processes (Augusto et al., 2016; PHE CRCE, 2018). Elevated benzo[a]pyrene concentrations were related to heavy traffic (i.e. lorries and trucks), which were also recorded at lichen sampling sites close to major roads across Manchester, suggesting particulate-bound (PM_2.5_ and PM_10_) impacts on lichen PAH concentrations (Augusto et al., 2016; Mastral et al., 2003; Slezakova et al., 2010). However, highest benzo[a]pyrene concentrations at green space locations suggesting additional PAH sources related to the specific surrounding. For instance, both sampling locations are in proximity (< 500 m) to railway lines, and PAH concentrations are potentially related to diesel and diesel electric locomotives, which may produce large amounts of black smoke and may be a significant source of PAHs (European Comission, 2001). Moreover, urban vegetation can impact on air quality by influencing deposition and dispersion of airborne pollutants (Janhäll, 2015; Kumar et al., 2019). Hence, contribution of PAH-containing particulate to the lichen PAH profile is suggested.

Overall, PAH concentrations across Manchester were found lower (Table S14), compared to other lichen biomonitoring studies undertaken in Portugal, India, Spain, France, Venezuela and Mexico (Augusto et al., 2016; Augusto et al., 2009; Bajpai et al., 2013; Blasco et al., 2006, 2007, 2011; Fernàndez et al., 2011; Guidotti et al., 2003; Kodnik et al., 2015; Puy-Alquiza et al., 2016; Shukla & Upreti, 2009). However, these studies were undertaken between 2006 and 2016, and recorded pollution levels may be linked to local emission sources, and study sites may now exhibit different pollutant patterns, e.g. launched low-emission zone and/or different traffic composition (i.e. electric and hybrid vehicles). Lower PAH concentrations across Manchester suggest minor PAH emissions, which is in accordance with emission reductions reported by Meijer et al. (2008). However, transport was reportedly the major source of PAHs across the UK, accounting for 65% of total PAH emissions (in 2005; Meijer et al., 2008). Moreover, Napier et al. (2008) reported PAH emissions from cars in the UK, being primarily related to oil losses, exhaust emissions, tyre erosion and brake wear, with regard to traffic flow. Traffic flows, especially travelling speed in the city centre of Manchester, have been found at ranges from 0 to 20 miles per hour (mph) during AM and PM peaks, suggesting additional vehicle-related (diesel and gasoline) PAH emissions (Highway Forecasting & Analytical Services, 2015; Napier et al., 2008).

Albeit potentially continued reduction of atmospheric PAHs, the lichen biomonitoring approach applied in this study still suggests deteriorated air quality across Manchester is primarily linked to vehicular emissions (i.e. 4-ring PAHs) and local sources (i.e. 5- and 6-ring). Indeed, lichen PAH profiles and individual concentrations recorded for Manchester suggest a predominance of local sources (Blasco et al., 2008; Satya et al., 2012; Shukla & Upreti, 2009) that warrant further investigation, e.g. using additional monitoring programmes to assess spatio-temporal variability of PAHs across Manchester. For instance, extending the lichen sampling approach could provide improve spatial resolution of PAH pollution across Manchester, that could be combined with a passive sampling approach to ‘translate’ lichen PAH loadings into atmospheric concentrations (Augusto et al., 2013).

### Spatial variability of PAH concentrations across Manchester

A lichen biomonitoring approach was capable to detect varying concentrations of individual PAHs across different land-uses and sampling locations across Manchester. Grouped data analysis showed significant (*p* < 0.05) differences between LMWs (2- and 3-ring PAHs) and road distance groups (Fig. S3), which was also found for individual PAHs, i.e. phenanthrene and pyrene. Pyrene is associated with combustion processes (e.g. fossil fuels, Blasco et al., 2006), whereas phenanthrene was found to be a major constituent in UK air (in 2005), and emissions are primarily related to motorised traffic, notably diesel trucks, lubricant oil and exhaust emission (Blasco et al., 2006; Meijer et al., 2008; Napier et al., 2008). Moreover, Charron et al. (2019) reported a relationship between NO_x_ and PAHs fluoranthene and pyrene. Within Manchester, about 80% of NO_x_ emissions are related to diesel vehicles, and elevated NO_2_ concentrations have been reported for Manchester city centre (Niepsch et al., 2021; Regan, 2018). Furthermore, 40% of licensed cars (about 12.7 million) in the UK are diesel cars (DfT, 2017b), hence suggesting vehicular emissions as primary source of PAHs in urban environments.

Spatial distribution of PAHs in air is also dependent on factors such as the size of particles they are adsorbed to, their hydrophilic character and the nature of the emission source (point or non-point) (Augusto et al., 2009). For lichens, the symbiotic algae content is a key factor for gas-phase PAH accumulation in lichens, which can also affect lichen photosynthesis, e.g. elevated fluoranthene (Augusto et al., 2015; Kummerová et al., 2006, 2007). However, environmental changes influence lichen morphology, physiology, chemistry and accumulation of pollutants (Nimis et al., 2002; Upreti et al., 2015) that need further investigation. Investigating lichen algae contents prior to extraction could inform about potential toxic effects of PAHs and accumulation potentials in (different) lichens.

In this study, the foliose (‘leaf-like’) lichen *X. parietina* (and *Physcia* spp.,* N* = 3; Table S12) were used, and species-specific accumulation abilities and sensitivity to PAH toxicity have been reported (Augusto et al., 2015; Kummerová et al., 2006, 2007). As primarily *X. parietina* samples were analysed for PAHs (and metals), species-specific impacts by atmospheric pollutants (i.e. PAHs) on lichen vitality are suggested. This is an important consideration, when using lichens as biomonitors and when comparing (urban) atmospheric PAH pollution, i.e. it is recommended to use only one particular lichen species. Additionally, lichens were sampled within a period of 5 months (during dry days/periods) to minimise variation of PAHs (Forbes, 2015). Indeed, lichens absorb contaminants (and nutrients) more or less constantly throughout their lifecycle (Blasco et al., 2006), and they are long-living organisms and thus integrate atmospheric pollutants over time, allowing to relate low levels of pollutants with long-term chronic effects on health (Augusto et al., 2007, 2013). However, in order to ensure temporal (and spatial) representation, long-term measurements and continuous sampling at a large number of sites are required (Shukla et al., 2014).

Overall, a lichen biomonitoring approach provides a useful tool to identify spatial variability of PAHs, aiding to identify areas of deteriorated air quality. Interestingly, elevated PAH concentrations might not only be limited to the city centre area of Manchester, but also at locations further away from major roads, e.g. green-spaces, due to potential influences from the particular surrounding. Linking PAH concentrations with PAHs in soils (around the sampling sites), active/passive monitoring of ambient concentration and measurements of PM-PAH concentrations could further improve spatial assessment.

### Source apportionment and human health risk assessment for lichen-derived airborne metal concentrations

**Fig. 6:**
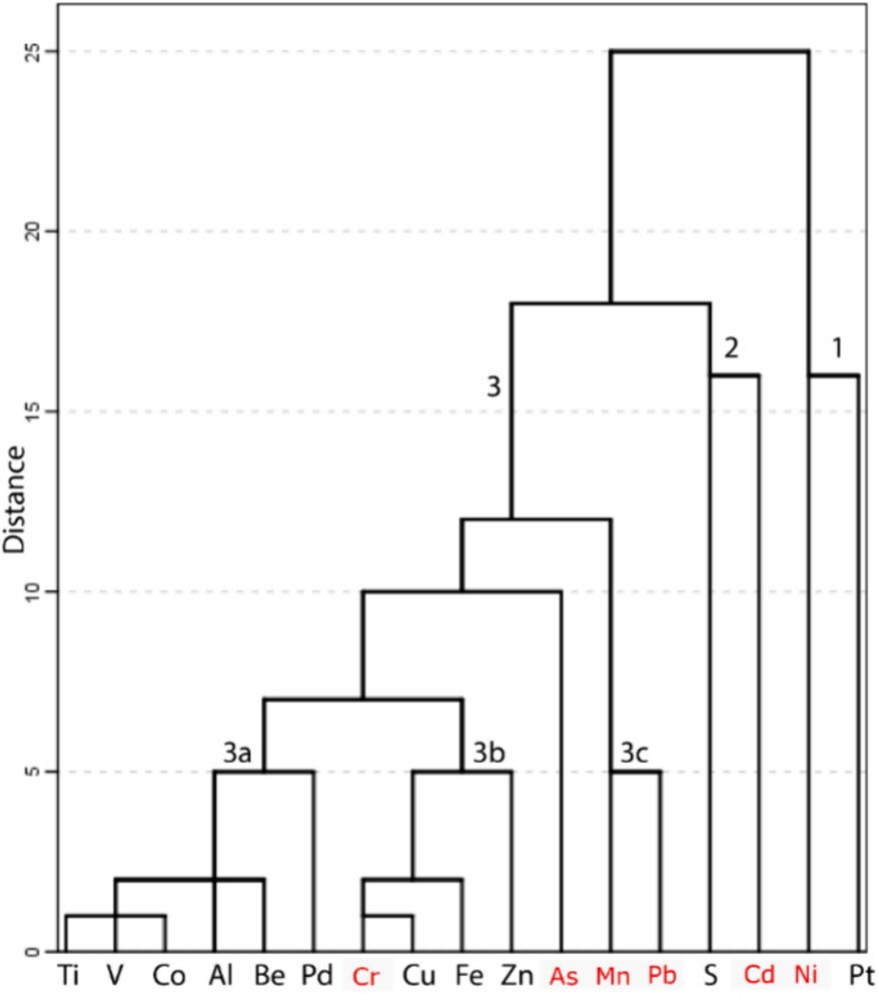
Dendrogram of 17 elements in samples of *X. parietina* (using complete linkage and squared Euclidian distance; Bozkurt, 2017) sampled across Manchester city centre (Fig. 1); shorter distances between elements illustrate higher similarity, further distances illustrate dissimilarities, target metals are highlighted in red

For cluster analysis, all 17 analysed elements were included to analyse potential relationships and aid identification of potential sources (Fig. 6), revealing three main clusters (Fig. 6), with Ni in cluster one, Cd in cluster 2, whereas As, Cr, Mn and Pb were located in cluster 3.

Nickel is part of alloys, plating, colours and catalysts, with emissions linked to industry and energy production (i.e. oil- and coal-burning power plants; ATSDR, 2005). Comparably, Cd in urban environments originates from tyre wear, vehicular abrasion and lubricating oils, alloys, paint pigments and plastics (ATSDR, 2012; ICdA, 2019; Taylor, 2006). Interestingly Cd, was clustered with sulphur (S), suggesting a similar source, i.e. ‘cadmium yellow’ used in car colours. Moreover, energy production, i.e. fuel combustion like coal and oil and manufacturing processes (i.e. iron and steel) are primary sources of Cd and S in the environment (DEFRA, 2017).

Manganese (Mn) and lead (Pb) were located in cluster 3c, which can be considered as traffic-related metals (Uluozlu et al., 2007). Nowadays, metal production and industrial lubricant combustion contain Pb (DEFRA, 2017), and Mn is used as a diesel fuel additive and could be further used as a tracer for railway wear (together with Cr and Fe) (Bukowiecki et al., 2007; Gehrig et al., 2007; Valotto et al., 2015; Wang et al., 2003). Mn was significantly (*p* < 0.001) positively correlated with Cr (*ρ* = 0.79; and Fe) indicating railway wear and vehicular sources. Moreover, Pb and Mn concentrations in road-deposited sediments in Manchester have been found to range between 71 and 594 µg/g (Pb) close to major traffic islands in the city centre of Manchester (Robertson & Taylor, 2007).

**Fig. 7:**
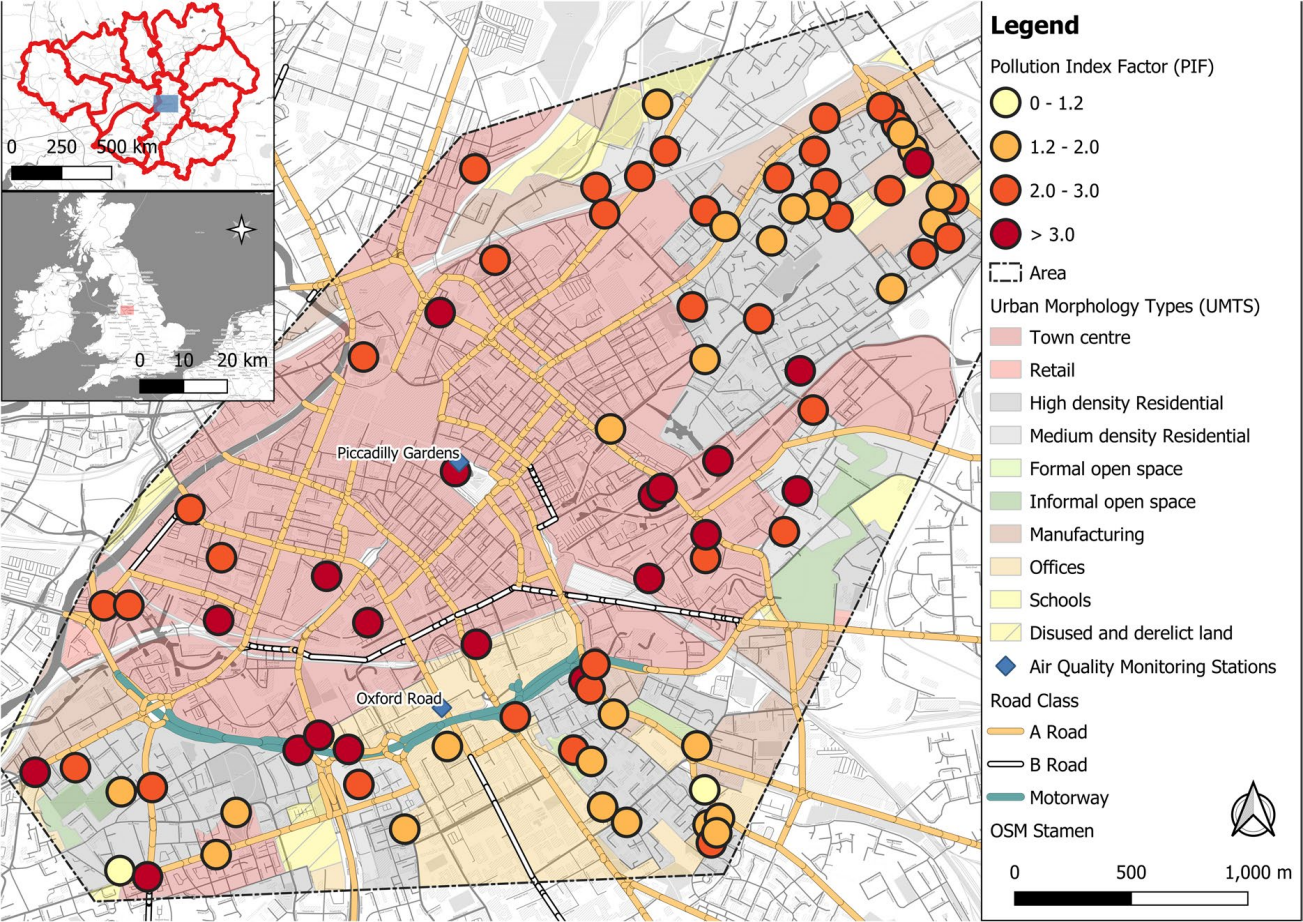
Pollution index factor (PIF) calculated for target metal concentrations recorded in *X. parietina* sampling locations (*N* = 87)

The overall pollutant load index for Manchester was calculated at 2.4, indicating ‘moderate’ pollution across the research area, whereas PLIs for sampling sites ranged between 1.0 and 6.4 (Fig. 7), with highest PLIs recorded at sampling sites classified as major road and town centre, and lower PFIs (< 3) in more residential surroundings south-east and northeast of the city centre. However, elevated PIF values in more residential surroundings (Fig. 7) suggest additional sources (e.g. domestic combustion) that may impact on ‘local’ air quality. Lowest PLIs in green spaces (PIF = 1.9) indicate beneficial impact of urban green and vegetation on airborne metal concentrations. Nonetheless, findings suggest poor air quality from airborne metals across Manchester, subsequently impacting on human health.

### Human health risk assessment for Manchester using lichen metal concentrations

Human health risk assessment for children (ΣPTEs HI: 3.23E^−02^) and adults (ΣPTEs HI: 1.40E^−02^) showed values below the threshold value (HI < 1) suggesting no negative effects on human health across the city centre of Manchester (Table 3).

**Table 3:** Hazard quotient (HI) and cumulative cancer risk (CCR) calculated for exposure assessment for adults and children, using (average) lichen metal concentrations for each element; missing values—no variable available to calculate for element; bold values represent calculated total HI and CCR

	Element	HI	CCR
Child	As	7.58E^−03^	3.20E^−06^
	Cd	5.68E^−04^	1.52E^−07^
	Cr	3.21E^−03^	4.07E^−06^
	Mn	1.23E^−03^	
	Ni	4.55E^−04^	
	Pb	1.68E^−02^	
	Cu	1.65E^−03^	1.09E^−04^
	Zn	8.69E^−04^	
	**ΣPTEs**	**3.23E**^−**02**^	**1.16E**^−**04**^
Adult	As	6.22E^−03^	2.80E^−06^
	Cd	4.43E^−04^	1.45E^−07^
	Cr	2.81E^−03^	3.88E^−06^
	Mn	4.15E^−04^	
	Ni	8.66E^−05^	
	Pb	3.53E^−03^	
	Cu	3.09E^−09^	1.95E^−05^
	Zn	1.77E^−04^	
	**ΣPTEs**	**1.40E**^−**02**^	**2.63E**^−**05**^

CCR values < 10^−6^ indicate low risk, 10^−6^ – 10^−4^ medium risk and > 10^−4^ a potentially severe risk (Khodadadi et al., 2023; Maghakyan, 2016; Singh et al., 2018). For Manchester, a medium carcinogenic risk for adults (2.63E^−05^) and children (1.16E^−04^) was found (Table 3), suggesting that one in every 37,962 adults or one in every 8586 child, respectively, has a probability to develop cancer within Manchester city centre. Such findings are particularly important, when considering that Manchester’s city centre belongs to the most densely populated areas across Manchester (Manchester City Council, 2018). Hence, illustrating the necessity to further evaluate and address poor urban air quality.

### Diagnostic ratios to fingerprint PAH sources and human health risk assessment related to PAH exposure across Manchester

PAH diagnostic ratios were used to investigate and identify potential pollutant sources across Manchester (Table S15). For instance, a ratio < 0.10 for ANT/(ANT + PHE) indicated petrogenic sources (44% of sites), whereas pyrogenic sources (ratio > 0.10) were identified for 56% of analysed sites. PHE/ANT alone suggests vehicular emissions as major cause of pollution at majority of sites (83%), which was further supported by FLT/PYR ratios < 1 and FLT/(FLT + PYR) ratios between 0.4 and 0.5 that can be used as indicators for vehicular emissions and fossil fuel combustion (e.g. gasoline, diesel and crude oil; (Augusto et al., 2016). Interestingly, PAH_comb_/PAH_total_ showed ratios > 0.7 for all analysed sites, suggesting combustion processes as major PAH sources across Manchester. In contrast, a BaA/(BaA + Chry) ratio < 0.2 showed petrogenic sources for 85% of analysed sites. Only four sites showed pyrogenic sources, using BaA/(BaA + Chry), which also found when using ANT/(ANT + PHE) for these sites (IDs 9, 7 and 15; Fig. S4).

Vehicular PAH emissions have been reported to vary with diurnal traffic patterns, i.e. gasoline and diesel vehicles (Marr et al., 2006). Within the UK, about 38 million vehicles are licensed, of which 83% were cars (59% petrol and 40% diesel-powered; DfT, 2017b). Three lichen sampling sites showed ‘petrogenic’ sources (Fig. S4), when using different diagnostic ratios, which could be related to traffic density and flow at the sampling location. Petroleum-derived PAHs could explain ‘local sources’, as tyre particles, asphalt and lubricant oils are associated with local sources of PAHs (Blasco et al., 2011; European Comission, 2001). Therefore, potential variability of vehicular fleets (i.e. diesel and gasoline cars, LGVs and HGVs) could influence the lichen PAH profile. Traffic count data is primarily available for major roads (counted or estimated; DfT, 2017a), and site-specific traffic data was not available.

**Fig. 8:**
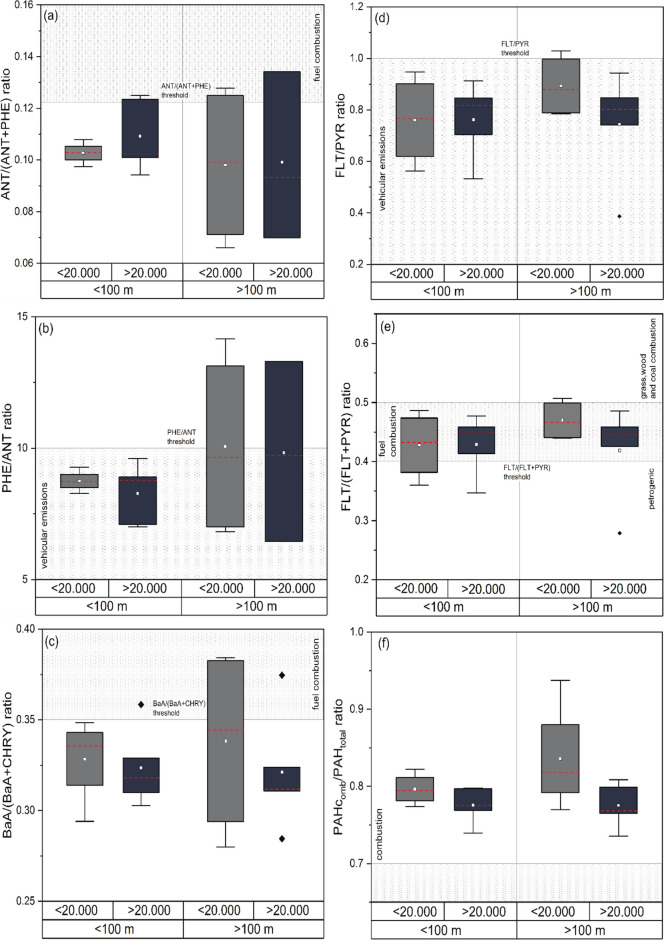
PAH diagnostic ratios and grouped distances to major road and traffic count data for (**a**) ANT/ANT + PHE, (**b**) PHE/ANT, (**c**) BaA/BaA + CHRY, (**d**) FLT/PYR and (**e**) FLT/PFLT + PYR and (**f**) PAHcomb/PAH total; displayed with diagnostic ratio thresholds and potential PAH origins

Photochemical reactions and chemical transformations after emissions (from any sources) can alter the pollutant composition of what was really emitted, and different accumulation abilities by different lichen species are considered as main issues, when using PAH ratios (Augusto et al., 2016). Indeed, diagnostic ratios suggest potential pollutant sources across Manchester, a clear distinction of potential PAH sources was not possible, and PAH diagnostic ratios were related to traffic count statistics and road distance groups (Fig. 8) to allow a safer interpretation of potential sources (Yunker et al., 2002). Findings suggest a complex mixture of PAHs in the urban environment of Manchester, from combustion (pyrogenic) and petrogenic (e.g. petroleum and oil) sources. However, approximately 90% of PAH emissions related to vehicular emission (light vehicular traffic in particular) that are distributed in the air in vapour- and particle-phase, and because of their stable molecular structure, PAHs undergo slow photochemical decomposition and degradation and thereby contribute to poor air quality in urban areas (Blasco et al., 2006; Nascimbene et al., 2014; Sarigiannis et al., 2015; Shukla et al., 2012).

It should be stated that these ratios require cautious interpretation, due to data obtained from 20 sampling sites (for *X. parietina*) only, and lichen-derived ‘PAH fingerprints’ have just been implemented recently (Augusto et al., 2016). For instance, extending the sampling towards more rural areas could aid detailed PAH source apportionment. Moreover, plant leaves have been reported to be a main sink of airborne PAHs and might intercept PAH accumulation in lichens within green spaces (Satya et al., 2012). This suggests that additional environmental compartments (i.e. tree leaves and needles) could be used to investigate PAH profiles across Manchester and identify potential interferences. Additional ratios (i.e. BaP/BaP + CHRY and Bbk/BbF among others) have been suggested that could be applied to (re-)define fingerprinting of PAH sources (Augusto et al., 2016). Nonetheless, PAH diagnostic ratios are a useful tool to evaluate potential origins of PAHs and support lichen-derived concentrations.

The carcinogenic potency across Manchester was dominated by known toxic PAHs benzo[a]pyrene and dibenz[a,h]anthracene, making up about 79% of the carcinogenic potential, which is comparable to reports from roadside soils and airborne particulates (Fang et al., 2002; Kumar et al., 2014). Benzo[b]- and benzo[k]fluoranthene (9%), indeno[1,2,3-cd]pyrene (6%) and benzo[a]anthracene (4%) are contributing 19% to the total carcinogenic potential. All other PAHs showed minor contribution to the total toxicity potential. Particulate-bound PAHs (≥ 4-rings) are primarily bound to the breathable fraction of particulates (PM_10_ and PM_2.5_), posing a significant threat to human health across Manchester, due to their elevated carcinogenic potential compared to low-molecular weight PAHs (Shukla et al., 2014; Slezakova et al., 2010). However, focussing on 16 EPA priority PAHs might strongly underestimate the toxic potential, by missing highly toxic PAHs, i.e. alkylated derivates, nitro- and oxy-PAHs of known toxicity that should be included in ambient monitoring programmes (Andersson & Achten, 2015; Lammel, 2015). For instance Samburova et al. (2017) analysed 88 gas and particle phase PAHs and reported that 16 EPA PAHs only contributed to 14% of the toxic equivalency (TEF), resulting in underestimation of potential health impacts.

The ILCR in Manchester was found at 1.455 × 10^−3^, indicating 1455 cancer cases could happen in one million people. Therefore, suggesting increased human health risks across Manchester from PAH exposure, particularly high-molecular PAHs. For Manchester, the ILCR was considerably higher than the US EPA limit of 1.5 × 10^−6^ (EPA, 2005). Manchester has the highest national average of cancer, which is about 1.6 times higher than the national average (Regan, 2018), indicating potential PAH impacts on human health across Manchester. Therefore, improving fine spatial detail of PAH concentrations across Manchester and incorporation of additional health relevant PAHs might benefit a more detailed human health assessment.

## Conclusion

This study aimed to assess the concentrations and spatial distribution of airborne metals and PAHs across Manchester (UK), identify potential sources and assess human health risk assessment, using a (high-resolution) lichen biomonitoring approach.

Lichens provide an easy-to-use and beneficial biomonitoring approach to assess and investigate major sources and spatial distribution of airborne metal and PAH concentrations and, hence, deteriorated air quality across an urban environment. Furthermore, lichen metal and PAH concentrations allowed evaluation of potential human health risks across Manchester, using different indices and exposure pathways. Such a lichen biomonitoring programme can be easily transferred to comparable urban environments to support automated air quality measurements and aid human health risk assessment studies across the UK (and other countries) for non-regularly monitored inorganic and organic air pollutants. Although sophisticated analytical techniques, to determine trace-levels of pollutants in lichen material, are required (i.e. resource and knowledge intensive), a lichen biomonitoring approach does not require additional equipment (i.e. automated sampling devices), (long-term) maintenance and access to power-sources. Hence, such information could be beneficial for local authorities, i.e. to identify areas of concern, and identify potential locations for ‘new’ or additional monitoring programmes.

Nonetheless, further research is required to translate lichen-derived airborne metal (e.g. particulate-bound) and PAH concentrations into atmospheric concentrations, i.e. by using a combined approach with active/passive ambient samplers is suggested. Additionally, lichen PAH diagnostic ratios need further investigation to aid ‘fingerprinting’ of (urban) pollution sources in more detail.

### Supplementary Information

Below is the link to the electronic supplementary material.

Supplementary file1 (DOCX 2296 KB)

## Data Availability

All data generated or analysed during this study are included in the article and its supplementary information.
