# Supplementary material for: Lichen biomonitoring to assess spatial variability, potential sources and human health risks of polycyclic aromatic hydrocarbons (PAHs) and airborne metal concentrations in Manchester (UK)
Source: Environ Monit Assess. 2024 Mar 18;196(4):379. doi: 10.1007/s10661-024-12522-4 (PMC11291577; doi:10.1007/s10661-024-12522-4)
Supplement: Supplementary file 1 — Supplementary file1 (DOCX 2296 KB) [file 10661_2024_12522_MOESM1_ESM.docx]

Lichen biomonitoring to assess spatial variability, potential sources and human health risks of polycyclic aromatic hydrocarbons (PAHs) and airborne metal concentrations in Manchester (UK)

Daniel Niepsch^1^, Leon J. Clarke^1^, Rhys G. Jones^2^, Konstantinos Tzoulas^1^, Gina Cavan^1^

^1^ Department of Natural Sciences, Faculty of Science & Engineering, Manchester Metropolitan University, M1 5GD, Manchester, UK

^2^ Waters Corporation, SK9 4AX, Wilmslow, UK

corresponding author: [Daniel.Niepsch@gmx.de](mailto:Daniel.Niepsch@gmx.de)

**Supplementary Information**

**Tab. S1:** Sites re-sampled for lichens for PAH determinations across Manchester (**Fig. 1b**) displayed with lichen N (wt%) and target metals (Pb, Cd, Cr, Ni, Mn and Zn) content, as well as rationale for sampling (MR = major road; GS = greenspace; RES = residential, with average annual daily traffic counts, where available; DfT, 2017); data values are displayed as three significant figures; * represents sites that have been sampled for both lichens, i.e. *X. parietina* and *Physcia* spp.

| Site ID | N  (wt%) | Pb  [µg/g] | Cd [µg/g] | Cr [µg/g] | Ni  [µg/g] | Mn [µg/g] | Zn  [µg/g] | Site rationale |
| --- | --- | --- | --- | --- | --- | --- | --- | --- |
| *Xanthoria parietina* (N=20) | | | | | | | |  |
| 1 | 3.18 | 43.1 | 0.181 | 11.6 | 6.10 | 61.0 | 171 | MR  15.600 |
| 2 | 2.18 | 13.7 | 0.0974 | 4.05 | 1.97 | 31.4 | 50.6 | GS |
| 3 | 3.04 | 13.8 | 0.139 | 3.41 | 1.82 | 31.7 | 77.5 | MR  25.700 |
| 4 | 3.41 | 13.9 | 0.189 | 4.32 | 2.58 | 36.1 | 74.6 | GS |
| 5* | 2.21 | 11.3 | 0.179 | 2.79 | 1.88 | 33.6 | 58.0 | RES |
| 6 | 2.86 | 10.8 | 0.118 | 3.78 | 2.66 | 32.3 | 47.9 | GS |
| 7 | 2.42 | 15.3 | 0.155 | 4.03 | 1.89 | 30.9 | 83.5 | GS |
| 8 | 3.60 | 14.4 | 0.147 | 3.89 | 1.84 | 35.8 | 60.4 | RES |
| 9 | 2.56 | 5.8 | 0.100 | 1.69 | 1.34 | 24.5 | 42.8 | RES |
| 10 | 3.35 | 22.9 | 0.147 | 6.67 | 2.83 | 50.6 | 199 | MR  59.400 |
| 11 | 2.50 | 29.5 | 0.159 | 6.42 | 2.67 | 44.7 | 112 | MR/RES  31.000 |
| 12 | 2.83 | 5.26 | 0.119 | 1.95 | 1.12 | 20.2 | 40.2 | RES |
| 13 | 2.82 | 20.2 | 0.136 | 7.39 | 5.60 | 41.8 | 108 | MR  32.000 |
| 14 | 2.71 | 9.23 | 0.313 | 2.57 | 1.99 | 32.4 | 72.5 | GS |
| 15* | 2.20 | N/A | N/A | N/A | N/A | N/A | N/A | RES |
| 16 | 2.68 | 9.99 | 0.0677 | 3.36 | 1.95 | 24.5 | 49.6 | MR/RES  31.000 |
| 17 | 2.35 | 8.39 | 0.104 | 1.54 | 2.11 | 25.9 | 64.9 | MR/RES  26.800 |
| 18 | 2.56 | 10.1 | 0.112 | 3.32 | 0.97 | 30.2 | 68.4 | GS |
| 19* | 2.39 | 15.4 | 0.113 | 2.79 | 1.95 | 37.2 | 86. | MR  26.700 |
| 20 | 3.06 | 14.4 | 0.112 | 2.74 | 3.20 | 44.1 | 98.1 | MR  10.200 |
| Physcia spp. (N=3) | | | | | | | |  |
| 5* | 1.92 | 8.74 | 0.147 | 1.94 | 1.70 | 27.3 | 81.9 | RES |
| 15* | 2.21 | 4.96 | 0.117 | 0.998 | 0.240 | 20.0 | 73.5 | RES |
| 19* | 2.32 | N/A | N/A | N/A | N/A | N/A | N/A | MR  26.700 |

**Tab. S2:** Microwave (CEM Mars Xpress 5) digestion programme (five step programme: temperature ramp up – digestion step I – temperature ramp up – digestion step II and cool down phase)

| Temperature ramp up – Step I |
| --- |
| Ramping temperature up to 90°C  Time: 10 minutes |
| Microwave digestion – Step II |
| Temperature: 90°C  Power: 600 W  Time: 5 minutes |
| Temperature ramp up – Step III |
| Ramping temperature up to 170°C  Time: 10 minutes |
| Microwave digestion – Step IV |
| Temperature: 170°C  Power: 1200W  Time: 10 minutes |
| Cool down phase – Step V |
| Time: 30 minutes |

**Tab. S3:** Elements determined by ICP-OES (Thermo Scientific iCap 6000 series) and ICP-MS (Agilent 7400), including the measured wavelengths (nm) and isotopes (latter indicated if analysed with helium collision cell by [He]) and lower limits of detection (LLD) ranges (min-max, in ng ml^-1^) for all analytical batches; key target metals shaded in grey

| ICP-OES | | | ICP-MS | | |
| --- | --- | --- | --- | --- | --- |
| Elements | **Analysed wavelength** [nm] | **LLD** (range: min- max [ng ml^-1^] | **Elements** | **Analysed isotope** | **LLD** (range: min- max [ng ml^-1^] |
| Aluminium (Al) | 167.0 | 0.005-0.035 | **Arsenic**  **(As)** | ^75^As [He] | 0.001-0.003 |
| Iron  (Fe) | 259.9 | 0.006-0.068 | **Beryllium**  **(Be)** | ^9^Be | 0.08-0.15 |
| Manganese (Mn) | 257.0 | 0.0001-0.003 | **Cadmium**  **(Cd)** | ^111^Cd [He] | 0.004-0.006 |
| Nickel  (Ni) | 231.6 | 0.001-0.012 | **Cobalt**  **(Co)** | ^59^Co [He] | 0.50-0.90 |
| Lead  (Pb) | 220.3 | 0.0006-0.012 | **Chromium**  **(Cr)** | ^52^Cr [He] | 0.007-0.014 |
| Sulphur  (S) | 182.0 | 0.005-0.014 | **Copper**  **(Cu)** | ^63^Cu [He] | 0.09-0.40 |
| Zinc  (Zn) | 206.2 | 0.008-0.077 | **Palladium**  **(Pd)** | ^105^Pd | 0.005-0.008 |
|  |  |  | **Platinum**  **(Pt)** | ^196^Pt | 0.002-0.02 |
|  |  |  | **Titanium**  **(Ti)** | ^47^Ti [He] | 0.006-0.01 |
|  |  |  | **Vanadium**  **(V)** | ^51^V [He] | 0.001-0.002 |

**Tab. S4:** Identification of 16 EPA PAHs by multiple reaction monitoring (MRM) and applied collision energies (V) and retention times (RT) using the GC-APCI instrument set-up.

| Compound | RT | MRMs (m/z) | Collision energy (V) |
| --- | --- | --- | --- |
| Naphthalene | 6.38 | 128.06 > 128.06 | 30 |
|  |  | 128.06 > 102.10 | 20 |
|  |  | 128.06 > 77.10 | 30 |
| Acenaphthylene | 8.43 | 152.08 > 150.08 | 30 |
|  |  | 152.08 > 126.08 | 27 |
|  |  | 152.08 >102.08 | 30 |
| Acenaphthene | 8.70 | 154.08 > 152.08 | 27 |
|  |  | 154.08 > 126.07 | 45 |
|  |  | 154.08 > 102.09 | 45 |
| Fluorene | 9.57 | 166.08 > 164.08 | 32 |
|  |  | 166.08 > 139.05 | 35 |
|  |  | 166.08 > 115.07 | 35 |
| Phenanthrene  Anthracene | 11.38  11.48 | 178.08 > 176.08 | 32 |
|  |  | 178.08 > 151.09 | 35 |
|  |  | 178.08 > 126.07 | 40 |
| Fluoranthene  Pyrene | 13.92  14.39 | 202.08 > 200.08 | 36 |
|  |  | 202.08 > 150.07 | 45 |
|  |  | 202.08 > 126.05 | 45 |
| Benz[a]anthracene  Chrysene | 17.17  17.23 | 228.09 > 226.09 | 50 |
|  |  | 228.09 > 200.10 | 50 |
|  |  | 228.09 > 176.08 | 50 |
| Benzo[b]fluoranthene Benzo[k]fluoranthene Benzo[a]pyrene | 19.49  19.55  20.11 | 252.09 > 250.09 | 45 |
|  |  | 252.09 > 224.08 | 55 |
|  |  | 252.09 > 200.08 | 55 |
| Dibenzo[a,h]anthracene | 22.22 | 278.11 > 276.11 | 40 |
|  |  | 278.11 > 250.10 | 55 |
|  |  | 278.11 > 226.10 | 50 |
| Indeno[1,2,3-cd]pyrene Benzo[ghi]perylene | 22.15  22.59 | 276.09 > 274.09 | 50 |
|  |  | 276.09 > 248.10 | 55 |
|  |  | 276.09 > 224.10 | 70 |
| Deuterated PAH standards | | | |
| Phenanthrene-d10 | 11.33 | 188.29 > 184.11 | 32 |
|  |  | 188.29 > 158.10 | 35 |
|  |  | 188.14 > 132.09 | 40 |
| Chrysene-d12 | 17.18 | 240.17 > 236.14 | 50 |
|  |  | 240.17 > 208.11 | 50 |
|  |  | 240.17 > 184.14 | 50 |
| Dibenzo[a,h]anthracene-d14 | 22.17 | 292.20 > 288.17 | 40 |
|  |  | 292.20 > 260.14 | 55 |
|  |  | 292.20 > 236.14 | 50 |

**Tab. S5:** Calibration standards for ICP-OES and ICP-MS measurements, as well as signal drift monitors, made from ESSLAB-910B for ICP-OES analysis and multi-elemental standards for ICP-MS (Esslab, 2017; Agilent Technologies, 2019)

| ICP-OES | | | |
| --- | --- | --- | --- |
| Calibration standard | Al, Ca, Fe, K, Na  [µg ml^-1^] | Cu, Mg, P, S  [µg ml^-1^] | Cd, Co, Cr_3_, Mn, Mo, Ni, Pb, Zn, As  [µg ml^-1^] |
| I | 0.1 | 0.05 | 0.02 |
| II | 0.16 | 0.08 | 0.032 |
| III | 1 | 0.5 | 0.2 |
| IV | 10 | 5 | 2 |
| V | 20 | 10 | 4 |
| VI | 40 | 20 | 8 |
| Signal drift monitor | | | |
|  | 2 | 1 | 0.4 |
| ICP-MS  Al, As, Be, Cd, Co, Cr, Cu, Fe, Mn, Ni, Pb, Pd, Pt, S, Ti, V, Zn [ng ml^-1^] | | | |
| I | 10 | | |
| II | 20 | | |
| III | 30 | | |
| IV | 40 | | |
| V | 50 | | |
| Signal drift monitor | | | |
|  | 10 | | |

**Tab. S6:** Measured (this study – mean values ± standard deviation; for all CRM measurements; N=23) and certified (and indicative) lichen CRM-482 elemental concentrations [µg g^-1^] for different acid digestion methods (Quevauviller et al., 1996; Baffi et al., 2002)

| Element | Measured values (HNO_3_ digestion – this study) N=23 | Certified values  (HNO_3_/HF digestion)^(a)^ | Measured values  (HNO_3_/H_2_O_2_ digestion)^(b)^ | Accuracy* (%)  (a)     (b) | |
| --- | --- | --- | --- | --- | --- |
| Al | 635.32 ± 81.51 | 1103 ± 24 | 592 ± 43 | 58 | 107 |
| As | 0.70 ± 0.05 | 0.85 ± 0.07 | 0.88 ± 0.44 | 83 | 80 |
| Be | 0.02 ± 0.002 | N/A | 0.002 ± 0.001 | N/A | 1030 |
| Cd | 0.45 ± 0.10 | 0.56 ± 0.02 | 0.56 ± 0.02 | 81 | 81 |
| Co | 0.27 ± 0.04 | 0.32 ± 0.03^†^ | 0.25 ± 0.01 | 85 | 109 |
| Cr | 2.09 ± 0.34 | 4.12 ± 0.15 | 2.20 ± 0.51 | 51 | 95 |
| Cu | 6.88 ± 2.32 | 7.03 ± 0.19 | 6.49 ±0.69 | 98 | 106 |
| Fe | 629.49 ± 37.45 | 804 ± 160^†^ | 688 ± 54 | 75 | 92 |
| Mn | 26.19 ± 0.94 | 33.0 ± 0.5^†^ | 27.1 ± 0.98 | 79 | 97 |
| Ni | 2.11 ± 0.25 | 2.47 ± 0.07 | 2.27 ± 0.05 | 85 | 93 |
| Pb | 38.08 ± 0.96 | 40.9 ± 1.4 | 34.8 ± 1.1 | 93 | 108 |
| Pd | 0.02 ± 0.01 | N/A | N/A | N/A | N/A |
| Pt | 0.003 ± 0.004 | N/A | N/A | N/A | N/A |
| S | 1829.65 ± 59.11 | 2166 ± 292^†^ | N/A | 85 | N/A |
| Ti | 11.29 ± 1.17 | 34.2 ± 1.1^†^ | 16.30 ± 2.6 | 33 | 69 |
| V | 2.71 ± 0.45 | 3.74 ± 0.61^†^ | 3.11 ± 0.19 | 72 | 87 |
| Zn | 97.32 ± 5.54 | 100.6 ± 2.2 | 93.5 ± 4.11 | 97 | 104 |
| ^(a)^ certified concentrations for CRM-482 and ^(b)^ values measured by Baffi *et al.* (2002)  * accuracy presented for comparison of this study’s concentrations with both digestion methods: first value = comparison to HNO_3_/HF digestion; second value = comparison to HNO_3_/H_2_O_2_ digestion  ^†^ indicative value | | | | | |

**Tab. S7:** Average recovery rates (%), LODs and LOQs (in pg – picogram) for 16 EPA PAH compounds determined in spiked lichen samples (N=4, TCL PAH mix) and standard solutions.

| Compound | Recovery (%) | LOD / LOQ  [in pg] | | Compound | Recovery (%) | LOD / LOQ  [in pg] | |
| --- | --- | --- | --- | --- | --- | --- | --- |
| Naphthalene | 58 | 0.16 | 0.50 | Benzo[a]anthracene | 103 | 0.10 | 0.29 |
| Acenaphthylene | 115 | 0.77 | 2.35 | Chrysene | 74 | 0.62 | 1.86 |
| Acenaphthene | 118 | 0.30 | 0.92 | Benzo[b]fluoranthene | 135 | 0.94 | 2.85 |
| Fluorene | 76 | 0.23 | 0.70 | Benzo[k]fluoranthene | 74 | 0.91 | 2.76 |
| Phenanthrene | 66 | 0.43 | 1.31 | Benzo[a]pyrene | 154 | 0.92 | 2.78 |
| Anthracene | 62 | 0.63 | 1.90 | Indeno[1,2,3-cd]pyrene | 80 | 0.95 | 2.87 |
| Fluoranthene | 118 | 0.44 | 1.33 | Dibenzo[a,h]anthracene | 66 | 0.59 | 1.78 |
| Pyrene | 117 | 0.43 | 1.31 | Benzo[ghi]perylene | 67 | 0.39 | 1.19 |


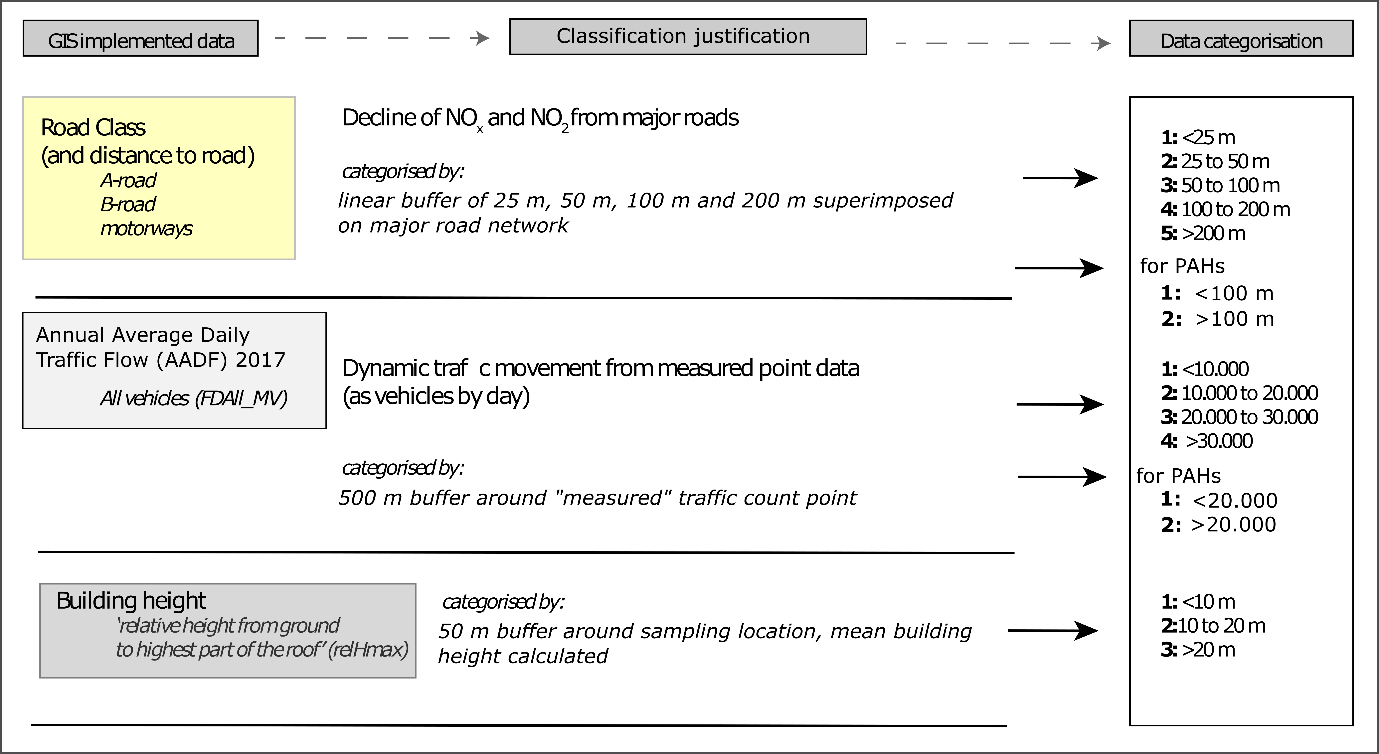


**Fig. S1:** Open-source data (road class data, AADF 2017 and building height; (DfT, 2017; Digimap - Ordnance Survey, 2017; UK Department of Transport, 2012) used for GIS-based categorisation of data and justification to evaluate the influence of urban factors on lichen-derived metal and PAH concentrations

**Tab. S8:** Descriptive statistics (mean ± 1x SD, for normal distributed data) for metal concentrations (in µg g^-1^; determined by ICP-OES and ICP-MS) in *Xanthoria parietina* samples collected from a rural area; As, Pb and Zn concentrations were not normally distributed and their average concentrations are expressed as medians

| Element | *X. parietina* (N=12) | |
| --- | --- | --- |
|  | Range (minimum – maximum) | Mean ± 1 standard deviation (or median **^#^**) |
| Aluminium (Al) | 175.35 – 535.11 | 321.18 ± 86.23 |
| Arsenic (As) | 0.23 – 1.76 | 0.44 **^#^** |
| Beryllium (Be) | 0.007 – 0.02 | 0.01 ± 0.002 |
| Cadmium (Cd) | 0.05 – 0.11 | 0.07 ± 0.02 |
| Cobalt (Co) | 0.08 – 0.25 | 0.15 ± 0.04 |
| Chromium (Cr) | 0.50 – 1.05 | 0.77 ± 0.15 |
| Copper (Cu) | 3.88 – 8.93 | 5.80 ± 1.49 |
| Iron (Fe) | 212.16 – 595.79 | 369.92 ± 94.75 |
| Manganese (Mn) | 22.23 – 52.06 | 33.67 ± 8.39 |
| Nickel (Ni) | 0.19 – 0.96 | 0.52 ± 0.21 |
| Lead (Pb) | 0.67 – 5.50 | 1.55 **^#^** |
| Palladium (Pd) | 0.01 – 0.02 | 0.02 ± 0.003 |
| Platinum (Pt) | 0.001 – 0.004 | 0.002 ± 0.0008 |
| Sulphur (S) | 2152.10 – 4730.08 | 3766.08 ± 789.38 |
| Titanium (Ti) | 2.89 – 11.20 | 6.69 ± 2.28 |
| Vanadium (V) | 0.43 – 1.02 | 0.76 ± 0.17 |
| Zinc (Zn) | 24.94 – 121.34 | 33.81 **^#^** |

**Tab S9:** Reference dose (RfD) and slope factor (SF) for potentially toxic elements: As, Cd, Cr, Ni, Pb, Cu and Zn used to calculate the hazard quotient (HQ) and the carcinogenic risk (CR) for human health risk assessment using the ingestion, inhalation and skin contact exposure pathways

| Element | Reference dose (mg/kg/day) | SF (mg/kg/day) |
| --- | --- | --- |
| Ingestion | |  |
| As | 3.00E^-04^ | 1.50E^00^ |
| Cd | 1.00E^-03^ | 5.01E^-01^ |
| Cr | 3.00E^-03^ | 0.50E^00^ |
| Mn | 1.40E^-01^ |  |
| Ni | 2.00E^-02^ |  |
| Pb | 3.00E^-03^ |  |
| Cu | 4.00E^-02^ | 1.70E^00^ |
| Zn | 3.00E^-01^ |  |
| Inhalation | |  |
| As | 3.10E^-04^ | 1.51E^01^ |
| Cd | 1.00E^-03^ | 6.30E^00^ |
| Cr | 2.86E^-03^ | 4.20E^01^ |
| Mn | 5.00E^-05^ |  |
| Ni | 2.06E^-02^ |  |
| Pb | 3.52E^-03^ |  |
| Cu | 4.02E^-02^ |  |
| Zn | 3.00E^-01^ |  |
| Skin contact/dermal exposure | |  |
| As | 1.23E^-04^ | 3.66E^00^ |
| Cd | 1.00E^-05^ | 2.00E^01^ |
| Cr | 6.00E^-05^ | 20.00E^00^ |
| Mn | 5.60E^-03^ |  |
| Ni | 5.40E^-03^ |  |
| Pb | 5.25E^-04^ |  |
| Cu | 1.20E^-02^ | 4.25E^00^ |
| Zn | 6.00E^-02^ |  |

**Tab. S10:** Toxic Equivalent Factors (TEF) for 16 EPA PAHs and ∑BaP_eq_. for each PAH

| PAH | Toxic Equivalence Factor (TEF) | ∑BaP_eq._ PAH |
| --- | --- | --- |
| Naphthalene | 0.001 | 0.0004 |
| Acenaphthylene | 0.001 | 0.0002 |
| Acenaphthene | 0.001 | 0.0001 |
| Fluorene | 0.001 | 0.0002 |
| Phenanthrene | 0.001 | 0.002 |
| Anthracene | 0.01 | 0.002 |
| Fluoranthene | 0.001 | 0.003 |
| Pyrene | 0.001 | 0.004 |
| Banz[a]anthracene | 0.1 | 0.08 |
| Chrysene | 0.01 | 0.02 |
| Benzo[b]fluoranthene | 0.1 | 0.12 |
| Benzo[k]fluoranthene | 0.1 | 0.03 |
| Benzo[a]pyrene | 1 | 1.02 |
| Dibenzo[a,h]anthracene | 5* | 0.29 |
| Indeno[1,2,3-cd]pyrene | 0.1 | 0.10 |
| Benzo[g,h,i]perylene | 0.01 | 0.009 |
| *a TEF of 1 appears to be appropriate for high doses of DahA but the TEF of 5 is considered more likely to be applicable to environmental exposure (Nisbet and LaGoy, 1992) | | |


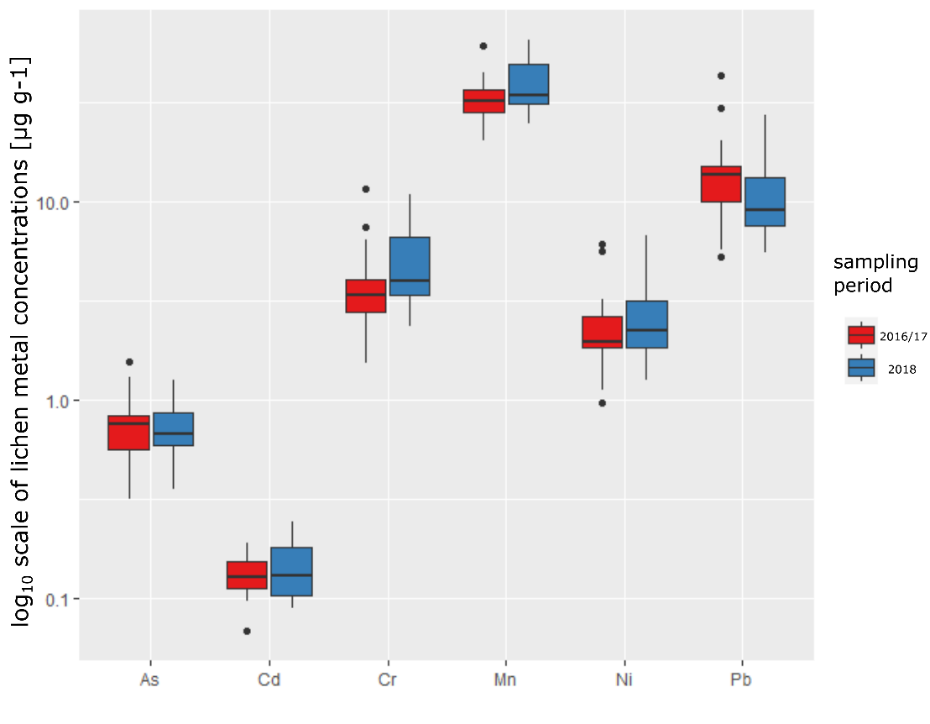


**Fig. S2:** Box-Whisker plots (25^th^ to 75^th^ percentile, displayed with mean – black line and outliers – black dots) of target metal concentrations in *X. parietina* for both sampling periods 2016/17 and 2018

**Tab. S11:** Wilcoxon test (due to different outcomes of Shapiro-Wilk normality test) for comparison of metal concentrations in *X. parietina* and *Physcia* spp. sampled from the same sites (N=15), displayed with significance levels *p<0.05and **p<0.01; key target metals presented in bold

| Element | Wilcoxon (two-tailed) |
| --- | --- |
| Aluminium (Al) | 0.0001** |
| Arsenic (As) | 0.01* |
| Beryllium (Be) | 0.0006** |
| Cadmium (Cd) | 0.02* |
| Cobalt (Co) | 0.0006** |
| Chromium (Cr) | 0.02* |
| Copper (Cu) | 0.005** |
| Iron (Fe) | 0.0006** |
| Manganese (Mn) | 0.04* |
| Nickel (Ni) | 0.37 |
| Lead (Pb) | 0.0003** |
| Palladium (Pd) | 0.72 |
| Platinum (Pt) | 0.12 |
| Sulphur (S) | <0.0001** |
| Titanium (Ti) | 0.0009** |
| Vanadium (V) | 0.125 |
| Zinc (Zn) | <0.0003** |

| Tab. S12: Correlation matrix of lichen-derived (*X. parietina*, N=84) metal concentrations | | | | | | | | | | | | | | | |
| --- | --- | --- | --- | --- | --- | --- | --- | --- | --- | --- | --- | --- | --- | --- | --- |
|  |  |  |  |  |  |  |  |  |  |  |  |  |  |  |  |
|  | |  | | **Mn** | | **Ni** | | **Pb** | | **Cr** | | **Cd** | | **As** | |
| Mn |  | Pearson's r |  | — |  |  |  |  |  |  |  |  |  |  |  |
|  |  | p-value |  | — |  |  |  |  |  |  |  |  |  |  |  |
|  |  | N |  | — |  |  |  |  |  |  |  |  |  |  |  |
| Ni |  | Pearson's r |  | 0.499 | *** | — |  |  |  |  |  |  |  |  |  |
|  |  | p-value |  | < .001 |  | — |  |  |  |  |  |  |  |  |  |
|  |  | N |  | 82 |  | — |  |  |  |  |  |  |  |  |  |
| Pb |  | Pearson's r |  | 0.793 | *** | 0.415 | *** | — |  |  |  |  |  |  |  |
|  |  | p-value |  | < .001 |  | < .001 |  | — |  |  |  |  |  |  |  |
|  |  | N |  | 84 |  | 82 |  | — |  |  |  |  |  |  |  |
| Cr |  | Pearson's r |  | 0.788 | *** | 0.626 | *** | 0.646 | *** | — |  |  |  |  |  |
|  |  | p-value |  | < .001 |  | < .001 |  | < .001 |  | — |  |  |  |  |  |
|  |  | N |  | 84 |  | 82 |  | 84 |  | — |  |  |  |  |  |
| Cd |  | Pearson's r |  | 0.531 | *** | 0.165 |  | 0.633 | *** | 0.407 | *** | — |  |  |  |
|  |  | p-value |  | < .001 |  | 0.138 |  | < .001 |  | < .001 |  | — |  |  |  |
|  |  | N |  | 84 |  | 82 |  | 84 |  | 84 |  | — |  |  |  |
| As |  | Pearson's r |  | 0.677 | *** | 0.352 | ** | 0.598 | *** | 0.661 | *** | 0.549 | *** | — |  |
|  |  | p-value |  | < .001 |  | 0.001 |  | < .001 |  | < .001 |  | < .001 |  | — |  |
|  |  | N |  | 84 |  | 82 |  | 84 |  | 84 |  | 84 |  | — |  |
| Note.: significance level: *p <.05, **p <.01, ***p <.001 | | | | | | | | | | | | | | | |
|  | | | | | | | | | | | | | | | |

**Tab. S13:** PAH concentration ranges [in ng g^-1^] recorded in *X. parietina* (N=20) and *Physcia* spp. (N=3) across Manchester; displayed with number of rings

| Rings | PAHs | *X. parietina* | *Physcia* spp. |
| --- | --- | --- | --- |
| 2-ring | Naphthalene (NAP) | 0.14-0.56 | 0.39-0.59 |
| 3-ring | Acenaphthylene (ACY) | 0.11-0.45 | 0.17-0.23 |
| 3-ring | Acenaphthene (ACN) | 0.06-0.55 | 0.10-0.15 |
| 3-ring | Fluorene (FLU) | 0.11-0.50 | 0.18-0.23 |
| 3-ring | Phenanthrene (PHE) | 1.32–7.55 | 2.08-2.77 |
| 3-rimg | Anthracene (ANT) | 0.12-1.05 | 0.28-0.33 |
| 4-ring | Fluoranthene (FLT) | 1.32–9.90 | 1.87-2.50 |
| 4-ring | Pyrene (PYR) | 1.84–9.63 | 3.10-3.44 |
| 4-ring | Chrysene (CHRY) | 0.64–4.79 | 0.87-1.16 |
| 4-ring | Benz[a]anthracene (BaA) | 0.31–2.95 | 0.31-0.57 |
| 5-ring | Benzo[b]fluoranthene (BbF) | 0.44–4.62 | 0.69-0.90 |
| 5-ring | Benzo[k]fluoranthene (BkF) | 0.10–1.44 | 0.19-0.53 |
| 5-ring | Benzo[a]pyrene (BaP) | 0.42–4.33 | 0.61-1.08 |
| 5-ring | Dibenzo[a,h]anthracene (DahA) | 0.01–2.47 | 0.02-0.04 |
| 6-ring | Indeno[1,2,3-cd]pyrene (IcdP) | 0.37–3.90 | 0.61-1.06 |
| 6-ring | Benzo[ghi]perylene (BghiP) | 0.37-3.90 | 0.51-0.80 |

**Tab. S14**: Overview of PAH concentrations recorded in lichens in urban and traffic-related pollution studies [shortened: excluding studies on landfill and mining sites etc.; Augusto et al., 2016]

| Lichen species | Topic and geographic area (and author) | Min/max concentrations [ng g^-1^] |
| --- | --- | --- |
| *Parmotrema hypoleucinum* | Urban, industrial, agricultural and forest – Sines (Portugal)  Augusto et al., 2009 | 91-872 (overall) |
| *Remototrachyna awasthii* | Urban – Mahableshwar City (India)  Bajpai et al., 2013 | Max. 62340 |
| *Parmelia sulcata* | Traffic – Somport tunnel (Spain and France) Blasco et al., 2006 | 910-1920 |
| *Parmelia sulcata* | Traffic – Aragon Valley (Spain)  Blasco et al., 2007 | 352-1652 |
| *Evernia prunastri* | Traffic – Aragon Valley (Spain)  Blasco et al., 2008 | 696-6240 |
| *Parmelia sulcata, Lobaria pulmonaria, Evernia prunastri, Ramalina farinacea, Pseudevernia farinacea, Usnea sp.* | Traffic – Aspe and Aragon Valley (France and Spain)  Blasco et al., 2011 | 238-6240 (overall) |
| *Xanthoria parietina* | Urban – Zaragoza City (Spain)  Domeño et al., 2006 | 340 |
| *Pyxine coralligera* | Urban – Caracas (Venezuela)  Fernàndez et al., 2011 | 240-9080 |
| *Pseudevernia furfuracea* | Traffic – Rieti, Latium (Italy)  Guidotti et al., 2003 | 36-375 |
| *Pseudevernia furfuracea* | Traffic – Viterbo, Latium (Italy)  Guidotti et al., 2009 | 168-395 |
| *Pseudevernia furfuracea* | Urban, industrial, agricultural and forest – Carnic pre-alps (Italy)  Kodnik et al., 2015 | 48-1576 |
| *Pseudevernia furfuracea* | Traffic – Dolomites, SE alps (Italy)  Nascimbene et al., 2014 | 186-2130 |
| *Xanthoparmelia mexicana* | Traffic – tunnels of Guanajuat City (Mexico)  Puy-Alquiza et al., 2016 | 522-3571 |
| *Rinodina sophodes* | Urban – Kanpur City (India)  Satya et al., 2012 | 189-494 |
| *Phaeophyscia hispidula* | Traffic – DehraDun City, Garhwal Himalayas (India)  Shukla and Upreti, 2009 | 3380-25010 |
| *Phaeophyscia hispidula, Phaeophyscia orbucilaris, Heterodermia angustiloba, Dimelaena oreina* | Traffic, urban – Garhwal Himalayas, Uttaranchal (India)  Shukla et al., 2010 | 683-33720 |
| *Pyxine subcinerea* | Urban, industrial and forest – Haridwar (India)  Shukla et al., 2012 | 1250-187300 |
| *Dermatocarpon vellereum* | Urban – Rudraprayag, Central Garhwal Himalayas (India)  Shukla et al., 2013 | 136-4961 |
| *Pseudevernia furfuracea* | Urban – Naples (Italy) and London (UK)  Vingiani et al., 2015 | 500  (85 in control samples) |


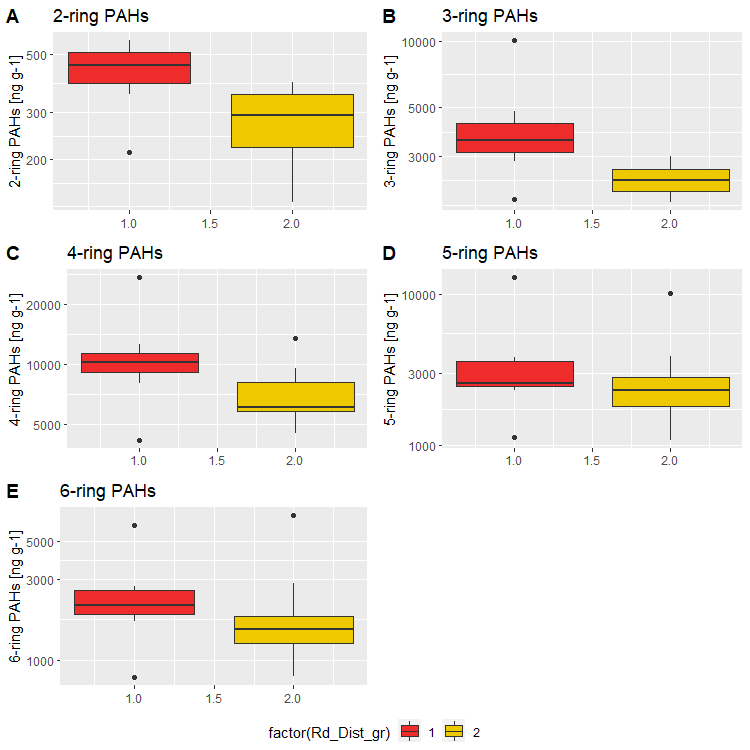


**Fig. S3:** Lichen PAH concentrations (by ring structure) in relation to major road distance groups (1: <500 m and 2: >500 m)

**Tab. S15:** Sampling site-IDs (for *X. parietina;* **Fig. 1**) displayed with analysed diagnostic PAH ratios to evaluate PAH sources/origin; N/A – not calculated due to no data for anthracene (ANT) at both sites

| ID | ANT/(ANT+PHE) | BaA/(BaA+CHRY) | FLT/PYR | PHE/ANT | FLT/(FLT+PYR) | PAH_comb_/PAH_total_ |
| --- | --- | --- | --- | --- | --- | --- |
| 1 | petroleum | petrogenic | vehicular emissions | vehicular emissions | fuel combustion | combustion |
| 2 | N/A | petrogenic | vehicular emissions | N/A | fuel combustion | combustion |
| 3 | petroleum | petrogenic | vehicular emissions | vehicular emissions | fuel combustion | combustion |
| 4 | combustion | pyrogenic | combustion | vehicular emissions | grass, wood and coal combustion | combustion |
| 5 | N/A | petrogenic | vehicular emissions | N/A | fuel combustion | combustion |
| 6 | petroleum | petrogenic | vehicular emissions | vehicular emissions | fuel combustion | combustion |
| 7 | petroleum | petrogenic | vehicular emissions | petrogenic | fuel combustion | combustion |
| 8 | petroleum | petrogenic | vehicular emissions | vehicular emissions | fuel combustion | combustion |
| 9 | petroleum | petrogenic | vehicular emissions | petrogenic | fuel combustion | combustion |
| 10 | combustion | petrogenic | vehicular emissions | vehicular emissions | petroleum | combustion |
| 11 | combustion | pyrogenic | vehicular emissions | vehicular emissions | fuel combustion | combustion |
| 12 | combustion | petrogenic | vehicular emissions | vehicular emissions | fuel combustion | combustion |
| 13 | combustion | petrogenic | vehicular emissions | vehicular emissions | fuel combustion | combustion |
| 14 | combustion | pyrogenic | vehicular emissions | vehicular emissions | fuel combustion | combustion |
| 15 | petroleum | petrogenic | vehicular emissions | petrogenic | fuel combustion | combustion |
| 16 | combustion | pyrogenic | vehicular emissions | vehicular emissions | fuel combustion | combustion |
| 17 | petroleum | petrogenic | vehicular emissions | vehicular emissions | fuel combustion | combustion |
| 18 | combustion | petrogenic | vehicular emissions | vehicular emissions | petroleum | combustion |
| 19 | combustion | petrogenic | vehicular emissions | vehicular emissions | fuel combustion | combustion |
| 20 | combustion | petrogenic | vehicular emissions | vehicular emissions | fuel combustion | combustion |


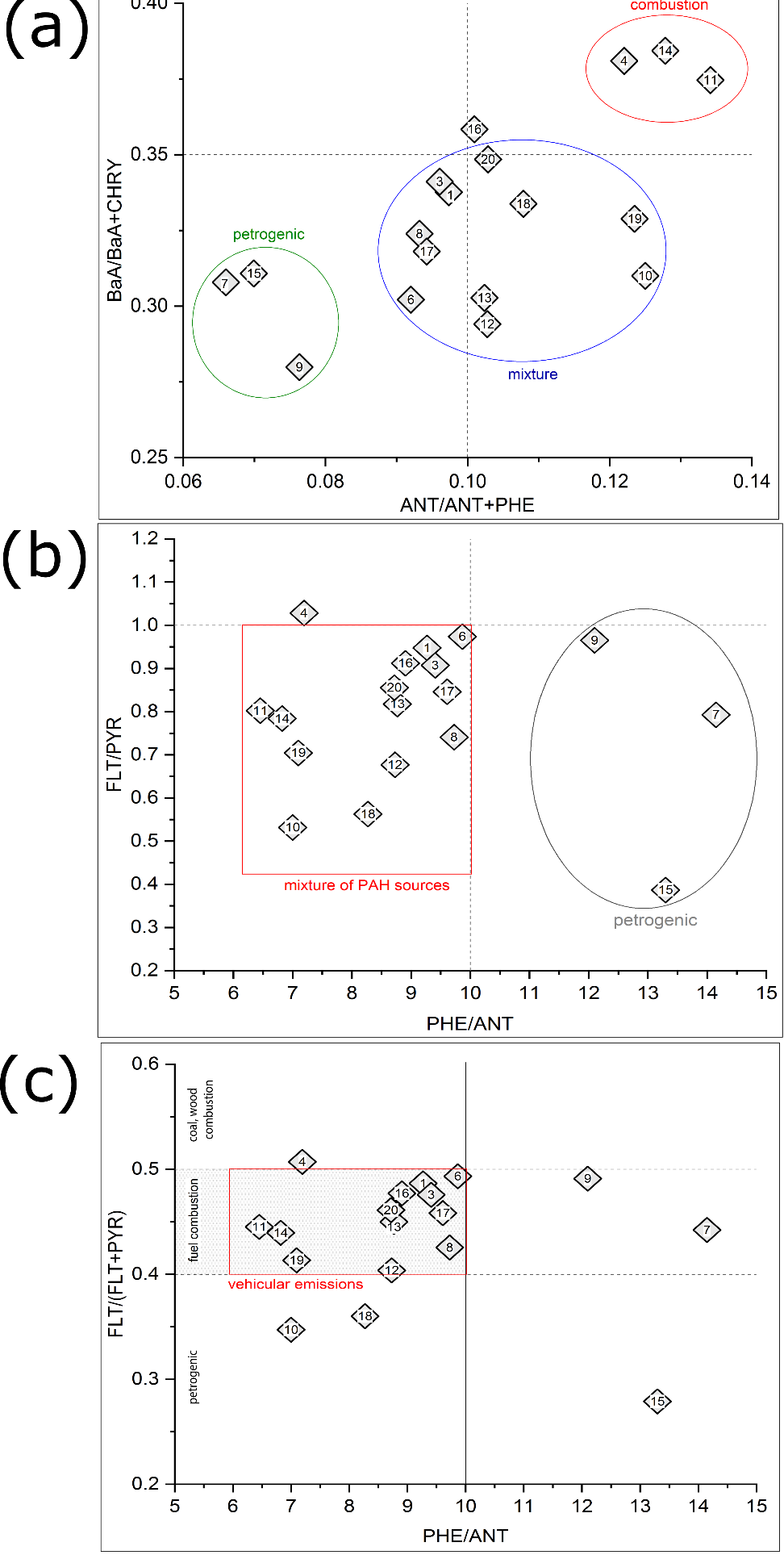

**Fig. S4:** Cross-plots of PAH diagnostic ratios for (a) ANT/ANT+PHE and BaA/BaA+CHRY, (b) FLT/PYR and PHE/ANT and (c) FLT/(FLT+PYR) and PHE/ANTrecorded at sampling sites (*X. parietina*, N=20); displayed with cluster of primary sources, i.e. petrogenic, petrogenic/combustion (mixture) and fuel combustion; dotted lines represent ratio thresholds

**References**

Augusto, S., Máguas, C., Matos, J., Pereira, M.J., Soares, A., Branquinho, C., 2009. Spatial Modeling of PAHs in Lichens for Fingerprinting of Multisource Atmospheric Pollution. Environ. Sci. Technol. 43, 7762–7769. https://doi.org/10.1021/es901024w

Augusto, S., Shukla, V., Upreti, D.K., Paoli, L., Vannini, A., Loppi, S., Nerín, C., Domeño, C., Schumacher, M., 2016. Biomonitoring of Airbourne Persistent Organic Pollutants Using Lichens, in: Tom, M., Vuković, G., Aničić Uroević, M. (Eds.), Biomonitoring of Air Pollution Using Mosses and Lichens: A Passive and Active Approach - State of the Art Research and Perspectives. Nova Science Publishers, Incorporated, pp. 2–41.

Bajpai, R., Karakoti, N., Upreti, D.K., 2013. Performance of a naturally growing Parmelioid lichen Remototrachyna awasthii against organic and inorganic pollutants. Environ. Sci. Pollut. Res. https://doi.org/10.1007/s11356-013-1583-3

Blasco, M., DomeÑo, C., Bentayeb, K., Nerín, C., 2007. Solid-phase extraction clean-up procedure for the analysis of PAHs in lichens. Int. J. Environ. Anal. Chem. 87, 833–846. https://doi.org/10.1080/03067310701381615

Blasco, M., Domeño, C., López, P., Nerín, C., 2011. Behaviour of different lichen species as biomonitors of air pollution by PAHs in natural ecosystems. J. Environ. Monit. 13, 2588. https://doi.org/10.1039/c0em00681e

Blasco, M., Domeño, C., Nerín, C., 2008. Lichens biomonitoring as feasible methodology to assess air pollution in natural ecosystems: Combined study of quantitative PAHs analyses and lichen biodiversity in the Pyrenees Mountains. Anal. Bioanal. Chem. 391, 759–771. https://doi.org/10.1007/s00216-008-1890-6

Blasco, M., Domeño, C., Nerín, C., 2006. Use of Lichens as Pollution Biomonitors in Remote Areas: Comparison of PAHs Extracted from Lichens and Atmospheric Particles Sampled in and Around the Somport Tunnel (Pyrenees). Environ. Sci. Technol. 40, 6384–6391. https://doi.org/10.1021/es0601484

DfT, 2017. Road traffic statistics [WWW Document]. URL https://www.gov.uk/government/publications/road-traffic-estimates-great-britain-jan-to-mar-q1-2014%5Cnhttps://www.gov.uk/government/collections/road-traffic-statistics

Digimap - Ordnance Survey, 2017. OS Building Heights (Alpha) [WWW Document]. URL https://digimap.edina.ac.uk/webhelp/os/data_information/os_products/os_building_heights.htm (accessed 12.11.18).

Domeño, C., Blasco, M., Sánchez, C., Nerín, C., 2006. A fast extraction technique for extracting polycyclic aromatic hydrocarbons (PAHs) from lichens samples used as biomonitors of air pollution: Dynamic sonication versus other methods. Anal. Chim. Acta 569, 103–112. https://doi.org/10.1016/j.aca.2006.03.053

Fernàndez, R., Galarraga, F., Benzo, Z., Màrquez, G., Fernàndez, A.J., Requiz, M.G., Hernàndez, J., 2011. Lichens as biomonitors for the determination of polycyclic aromatic hydrocarbons (PAHs) in Caracas Valley, Venezuela. Int. J. Environ. Anal. Chem. 91, 230–240. https://doi.org/10.1080/03067310903198478

Guidotti, M., Stella, D., Dominici, C., Blasi, G., Owczarek, M., Vitali, M., Protano, C., 2009. Monitoring of Traffic-Related Pollution in a Province of Central Italy with Transplanted Lichen Pseudovernia furfuracea. Bull. Environ. Contam. Toxicol. 83, 852–858. https://doi.org/10.1007/s00128-009-9792-7

Guidotti, M., Stella, D., Owczarek, M., De Marco, A., De Simone, C., 2003. Lichens as polycyclic aromatic hydrocarbon bioaccumulators used in atmospheric pollution studies. J. Chromatogr. A 985, 185–190. https://doi.org/10.1016/S0021-9673(02)01452-8

Kodnik, D., Candotto Carniel, F., Licen, S., Tolloi, A., Barbieri, P., Tretiach, M., 2015. Seasonal variations of PAHs content and distribution patterns in a mixed land use area: A case study in NE Italy with the transplanted lichen Pseudevernia furfuracea. Atmos. Environ. 113, 255–263. https://doi.org/10.1016/j.atmosenv.2015.04.067

Nascimbene, J., Tretiach, M., Corana, F., Lo Schiavo, F., Kodnik, D., Dainese, M., Mannucci, B., 2014. Patterns of traffic polycyclic aromatic hydrocarbon pollution in mountain areas can be revealed by lichen biomonitoring: A case study in the Dolomites (Eastern Italian Alps). Sci. Total Environ. 475, 90–96. https://doi.org/10.1016/j.scitotenv.2013.12.090

Nisbet, I.C.T., LaGoy, P.K., 1992. Toxic equivalency factors (TEFs) for polycyclic aromatic hydrocarbons (PAHs). Regul. Toxicol. Pharmacol. 16, 290–300. https://doi.org/10.1016/0273-2300(92)90009-X

Puy-Alquiza, M.J., Reyes, V., Wrobel, Katarzyna, Wrobel, Kazimierz, Torres Elguera, J.C., Miranda-Aviles, R., 2016. Polycyclic aromatic hydrocarbons in urban tunnels of Guanajuato city (Mexico) measured in deposited dust particles and in transplanted lichen Xanthoparmelia mexicana (Gyeln.) Hale. Environ. Sci. Pollut. Res. https://doi.org/10.1007/s11356-016-6256-6

Satya, Upreti, D.K., Patel, D.K., 2012. Rinodina sophodes (Ach.) Massal.: a bioaccumulator of polycyclic aromatic hydrocarbons (PAHs) in Kanpur City, India. Environ. Monit. Assess. 184, 229–238. https://doi.org/10.1007/s10661-011-1962-5

Shukla, V., Patel, D.K., Upreti, D.K., Yunus, M., 2012. Lichens to distinguish urban from industrial PAHs. Environ. Chem. Lett. 10, 159–164. https://doi.org/10.1007/s10311-011-0336-0

Shukla, V., Upreti, D.K., 2009. Polycyclic aromatic hydrocarbon (PAH) accumulation in lichen, Phaeophyscia hispidula of DehraDun City, Garhwal Himalayas. Environ. Monit. Assess. 149, 1–7. https://doi.org/10.1007/s10661-008-0225-6

Shukla, V., Upreti, D.K., Patel, D.K., Tripathi, R., 2010. Accumulation of Polycyclic Aromatic Hydrocarbons in some lichens of Garhwal Himalayas, India. Int. J. Environ. Waste Manag. 5, 104. https://doi.org/10.1504/IJEWM.2010.029695

Shukla, V., Upreti, D.K., Patel, D.K., Yunus, M., 2013. Lichens reveal air PAH fractionation in the Himalaya. Environ. Chem. Lett. 11, 19–23. https://doi.org/http://dx.doi.org/10.1007/s10311-012-0372-4

UK Department of Transport, 2012. Guidance on Road Classification and the Primary Route Network.

Vingiani, S., De Nicola, F., Purvis, W.O., Concha-Graña, E., Muniategui-Lorenzo, S., López-Mahía, P., Giordano, S., Adamo, P., 2015. Active Biomonitoring of Heavy Metals and PAHs with Mosses and Lichens: a Case Study in the Cities of Naples and London. Water, Air, Soil Pollut. 226, 1–12. https://doi.org/10.1007/s11270-015-2504-5
